# Bonding with a Benchtop UV Ozone Cleaner: A Practical Guide to Prepare a Microfluidic Chip

**DOI:** 10.3390/mi17091050

**Published:** 2026-09-02

**Authors:** Mirjam P. M. Poschmann, Lucas Holtorf, Igor Titov, Nahomy M. Lizarde, Thomas Strunskus, Martina Gerken

**Affiliations:** 1 Chair for Integrated Systems and Photonics, Faculty of Engineering, Kiel University, Kaiserstraße 2, 24143 Kiel, Germany; lho@tf.uni-kiel.de (L.H.); igti@tf.uni-kiel.de (I.T.); 2 Chair for Composite Materials, Faculty of Engineering, Kiel University, Kaiserstraße 2, 24143 Kiel, Germanyts@tf.uni-kiel.de (T.S.); 3 Kiel Nano, Surface and Interface Sciences (KiNSIS), Kiel University, Christian-Albrechts Platz 4, 24118 Kiel, Germany

**Keywords:** microfluidics, bonding, UV ozone treatment

## Abstract

This study describes a procedure for preparing microfluidic chips made from polydimethylsiloxane (PDMS) layers. It uses an Ossila UV ozone cleaner for surface activation instead of the more common oxygen plasma treatment. The process is limited to PDMS@PDMS bonding, and it can be used when a low-cost procedure for a small number of microfluidic chips is required or when an oxygen plasma etcher is unavailable. The challenges of this process arise from the slight hardening of the PDMS surface when it is activated for at least 70 min, which is necessary for reliable bonding. Activation of the surface is confirmed by contact angle measurements. In a static simulation and lab tests, it is demonstrated that damage resulting from this hardening in conjunction with careless handling of the microfluidic chip is mitigated by incorporating predetermined break structures and using tubes with an outer diameter that is smaller than the inlets. Additionally, pre-polymerized PDMS glue and PDMS seals are suggested to ensure that the tubes are properly sealed. A burst test in air was performed to demonstrate the stability of the bonding and gluing. IR and XPS measurements were performed to investigate surface and bulk changes due to the treatment. To demonstrate the concept, six microfluidic chips were prepared and tested, achieving flow rates of at least 170 µL/min at ±180 mbar. Bonding remains stable up to a pressure of approximately 428 ± 60 mbar.

## 1. Introduction

In recent decades, microfluidic devices have become a field of high interest due to their variability and adaptability, which ensures their use in a wide range of biological and chemical point-of-care analysis devices [1,2,3,4,5]. So-called ‘lab-on-a-chip’ devices enable test reagents to be combined within microchannel systems for various purposes, such as mixing, separating, filtering, carrying out chemical reactions and taking measurements, when combined with additional measurement compounds [6,7,8,9,10]. These systems are operated using, e.g., micro-pumps and valves [2,11,12]. The chips are made from various polymers, such as poly(methyl methacrylate), polystyrene and poly(dimethyl siloxane) (PDMS), as well as less common polymers for special applications. Production methods include injection molding, cutting, 3D printing and lithography [2,13,14,15]. These methods are used to create layers with one-sided open channel systems that are closed by bonding them to a substrate. Bonding is therefore a central process step in microfluidic chip fabrication, and multiple different methods have been described, e.g., applying tape or glue, using chemical or physical surface modifications or hot embossing [16,17,18].

The use of PDMS is one of multiple options and especially common in research laboratories and during the prototyping phase due to its optical transparency and fast preparation and integration of the chips [19,20]. Small batches of these chips are often produced using photolithography and soft lithography processes, but other molds are also used. In this process, a one-sided open channel system is created in a PDMS layer by mixing the pre-polymer and the curing agent, pouring the mixture into the individual mold and allowing it to polymerize at room temperature or an elevated temperature [21,22]. The PDMS layer is then sealed by bonding it to a substrate, such as glass or polymers [20,23,24]. This bonding is most often achieved by bringing the two layers into contact after they have been activated in oxygen plasma, as this is usually a highly reliable process [25]. However, we observed some challenges when working with an SI 100 by Sentech, as it is an older instrument used for multiple processes. We had to adapt our bonding protocol several times when issues occurred, such as smaller or larger leakages, different pressures in the gas supply line, hardware changes after maintenance work was performed or contamination of the chamber by other processes. These problems resulted in inconsistent bonding, so alternatives were tested simultaneously. One option is to use a PDMS/toluene mixture or pre-polymerized PMDS [26,27]. However, both methods result in low bonding strength. Additionally, the glue partially or fully blocks small channels, meaning it cannot be used for channels measuring 200 µm × 200 µm or smaller. Another possibility is a UV ozone treatment (UVO), which is a well-established method for substrate cleaning and surface functionalization [28]. Successful bonding was achieved using UV or UVO treatment alongside thermal bonding at temperatures 5–10 °C lower than the individual glass transition temperatures T_g_ or the Vicat softening point T_v_ for poly(methylmethacrylate) (T_g_ = 98 °C) [29,30], cyclic olefin copolymer (T_g_ = 105 °C) [30], polystyrene (T_v_ = 93 °C) and cyclic polyolefine (T_g_ = 70 °C) [31].

During UVO treatment of PDMS, oxygen dissociates at a wavelength of 184.9 nm, while ozone dissociates at 253.7 nm. The latter wavelength is absorbed by hydrocarbons, leading to their oxidation by reactive oxygen species [32]. This process involves removing the methyl groups from the PDMS surface and the bulk material close to the surface and the formation of a thin, glass-like, amorphous SiO_2_ layer. The thickness of this layer depends on the treatment time and lamp intensity. The treatment results in long-lasting hydrophilic behavior, which could be useful for certain applications [32,33]. However, the treatment results in slight hardening of the surface. Berdichevsky et al. [33] describe cracks forming when the device’s temperature exceeds 100 °C and the treatment time exceeds 30 min. Additionally, they describe a permanently reduced contact angle after 2 h treatment times, an improved wettability, electro-osmotic flow and lower adsorption of a rhodamine dye due to the hydrophilic surface. To bond the surface, the researchers in that study applied an additional thin PDMS film and used oxygen plasma bonding and not UVO bonding.

Yousuff et al. [34] compared the bonding strength of PDMS@glass and PDMS@PDMS samples bonded using UVO and oxygen plasma treatments. The bonding strength with UVO was higher in the case of PDMS@glass (314 ± 20 kPa) and lower in the case of PDMS@PDMS (95 ± 20 kPa) compared to oxygen plasma treatment (279 ± 20 kPa and 180 ± 20 kPa, respectively). The same observation was made regarding PDMS@PDMS bonding by Chen et al. [35] (0.78 ± 0.08 MPa vs. 2.34 ± 0.27 MPa). In some other studies, researchers utilize UVO bonding to prepare microfluidic systems, and we summarized the information concerning the bonding in the Supplementary Materials in Table S1 [36,37,38,39,40,41,42,43]. Treatment times ranging between 3 and 40 min and various post-treatments at elevated temperatures are used. However, the focus of all these studies is on the investigated devices rather than on the UVO bonding process itself. In most cases, different types of biosensing applications were investigated, and the microfluidic chips were used for containment or measurement [44,45,46]. In some cases, it was used for liquid control such as mixing, separation or actuation [47,48]. In one case, a peel strength of ~30 kPa and a maximum flow rate of 1500 µL/min were found [49]. However, there is very little information on the UVO bonding itself, so it is difficult to identify the reasons for the different conditions. We were unable to reproduce the results using our UV ozone cleaner (L2002A3-EUmodel by Ossila Ltd., Sheffield, UK), possibly due to differences in the instruments, geometries, intensities or other parameters.

Although the use of UVO bonding has been reported, to the best of our knowledge, no study has discussed the specific challenges, limitations and reproducibility of UVO bonding in detail, with a focus on microfluidic chip preparation. This is important for the further deployment of UVO bonding. In this study, we present a method for preparing PDMS-based microfluidic chips with our UV ozone cleaner by Ossila. We observed issues arising from the hardening of the PDMS surface and explored ways to address the most important factors in preparing a microfluidic chip: these were the optimization of bonding conditions, the mechanical stability in the context of a lower bonding strength and leakage prevention (see Figure S1, left). Finally, we demonstrate the working range by preparing six microfluidic chips and showing their functionality and limitations.

## 2. Materials and Methods

The Si-wafer 4P0/5-10/525 ± 20/SSP/TTV < 5 (Sigert Wafer), GM1075 photoresist (Gersteltec, Pully, Switzerland), 1-Methoxy-2-propylacetat PGMEA (Carl Roth, Karlsruhe, Germany), polydimethylsiloxane PDMS (Sylgard™ 184 and Curing Agent by Dow Corning, Midland, MI, USA), polished float glass (Präzisions Glas & Optik GmbH, Iserlohn, Germany), spin coater Opti Spin ST22P (Robotechnik Europe GmbH, Singen, Germany) and WS-650Mz-8NPPB (Laurell Technologies corporation, Landsdale, PA, USA), mask aligner MA6/BA6 (Süss MicroTec Lithography GmbH, Garching, Germany), plasma etcher SI 100 (Sentech, Berlin, Germany), UV ozone cleaner with emissions at 185 and 254 nm and with an intensity of 15 mW/cm^2^ at 185 nm (Ossila Ltd., Sheffield, UK), confocal microscope VK-X260-K (Keyence, Osaka, Japan), contact angle measurement system OCA 50 AF (Dataphysics Instrument GmbH, Filderstadt, Germany), profilometer DektakPro (Bruker, Billercia, MA, USA), FTIR instrument Invenio R (Bruker, Billercia, MA, USA), XPS PREVAC-Multichamber XPS System equipped with an Al K_α_ X-ray source (1486.6 eV) operated at 300 W power and a hemispherical analyzer operated with a 3 mm spot size, pressure sensor ABPDRRV015PDAA5 (Honeywell International Inc., Tempe, AZ, USA), stepper pump RP-QIIICP5S-P18A-DC3VS (Takasago Electric, Inc., Fukuoka, Japan), DC pump RP-QIIIA1.5S-3Z-DC3V (Takasago Electric, Inc., Fukuoka, Japan) actuated with an electronic board and a Raspberry PI, as described before [50], are used.

### 2.1. Photolithography

Prior to usage, the wafer was cleaned 5 min in acetone, isopropanol and water, each in an ultrasonic bath, and dehydrated for 5 min on a hot plate at 150 °C. The surface activation was performed in an oxygen plasma etcher for 6 min at 0.3 mbar and 300 W. Afterwards, GM1075 was spin-coated on the wafer using, for the first step, a ramp of 100 rpm/s up to 1900 rpm and holding that for 110 s, as well as a second step, in which spinning was increased for 1 s to 2300 rmp with 400 rpm/s. Afterwards, the wafer was placed on a horizontally leveled hot plate at 40 °C for 30 min. Then, a heat ramp of 4 °C/min was used to reach 120 °C, and we held that temperature for 2 min. Afterwards, the wafer was cooled down to room temperature and exposed to UV light (I-line) at 200 mJ/cm^2^. Post-exposure baking was performed for 2 min at 65 °C and 25 min at 95 °C. After one day of waiting time, the wafer was developed in PGMEA for 4.75 min. The structure thickness was confirmed with a confocal microscope.

### 2.2. Soft Lithography

A PDMS mixture with a 10:1 Sylgard™ 184:Curing Agent ratio was prepared in quantities of 20 or 30 g for PDMS layers with thicknesses of 2 mm and 3 mm, respectively. This mixture was then thoroughly mixed and degassed in a desiccator. The wafer master was placed in an aluminum foil mold, into which the PDMS mixture was poured and degassed again. The PDMS was then cured for 20 min at 130 °C on a hot plate. Afterwards, it was peeled off the wafer and cut into pieces. In the case of the samples for the bonding optimization, a blank wafer was used, and the created PDMS layer was cut into 1 × 1 cm pieces.

### 2.3. Contact Angle Measurement

Contact angle measurements were taken on PDMS and glass carriers that were treated in the UVO cleaner for 10, 20, 30, 40, 50, 60 or 70 min. Samples treated for 10, 30, 50 or 70 min were treated together, as not all samples fit in the instrument chamber. Similarly, samples treated for 20, 40 or 60 min were treated together. Measurements were taken a few min after the treatment and approximately 24 h afterwards. A total of 3 µL of deionized water was applied at a rate of 10 µL/s to the samples in three different positions. Each time, three droplets were measured three times using tangent leaning.

### 2.4. UVO Bonding

**Figure 1:**
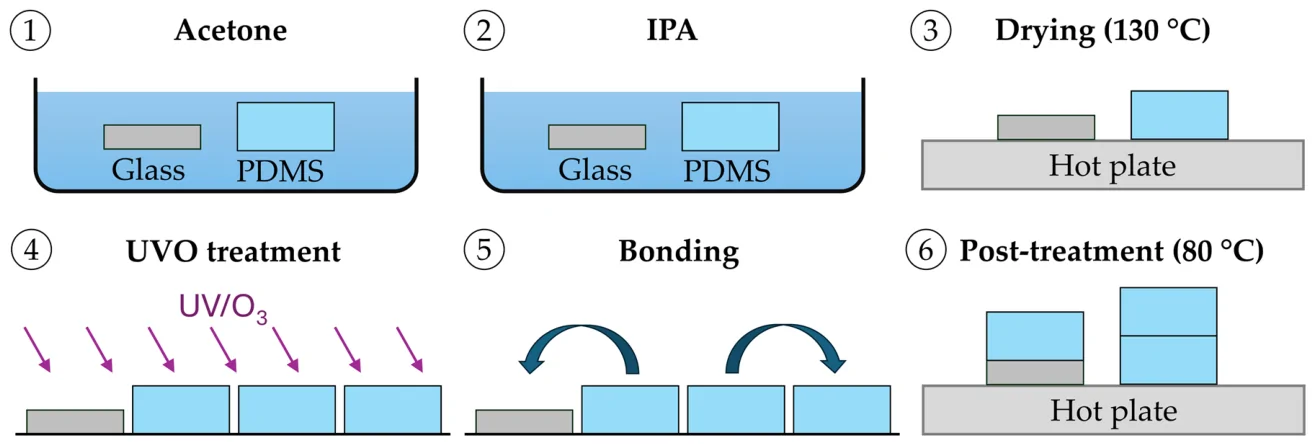
Schematic illustration of the bonding process.

PDMS sheets, 2–3 mm thick, were cut into 1 cm × 1 cm pieces. These were cleaned in an ultrasonic bath with acetone for 1 min each and then dried on a hot plate at 130 °C for 5 min. The glass pieces (2.5 cm × 2.5 cm) were cleaned in an ultrasonic bath with acetone and IPA for 5 min and then dried on a hot plate at 130 °C for 15 min. Most times, seven different positions were tested in the measurement to average out mistakes and confirm homogeneous activation within the chamber (see Supplementary Materials Figure S1, right). If the samples were to be treated equally, activation in the ozone cleaner was performed in parallel for the same amount of time. If different times were used, the glass was usually treated for the difference in time between the two samples. After this, the two samples were activated together for the remaining time. The samples were pressed together within the first minute after activation using only slight pressure. A post-treatment at 80 °C was then performed for 1 h. The whole process is illustrated in Figure 1.

UVO treatments lasting between 10 and 90 min were tested, and the positions of the samples were noted to determine whether the treatment was homogeneous across the entire chamber. After 20–24 h, the samples were peeled off to test the bonding.

### 2.5. Peel Test

There are several methods to determine the bonding strength [25]. We selected a manual peel test for the optimization of the bonding conditions because it does not require any special sample preparation or equipment and is easily adaptable to different sample sizes. Samples were manually peeled, and the peelable area was determined.

### 2.6. Predetermined Breaking Structures

**Figure 2:**
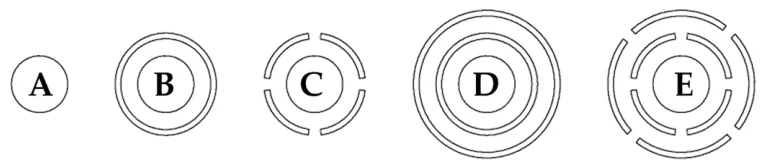
Schematic design of the standard inlet (**A**) and the four inlets with predetermined breaking structures (**B**–**E**). A more detailed version is given in Supplementary Materials Figure S4.

To investigate the mechanical stability of inlets and outlets, predetermined breaking structures were designed. A master wafer was prepared, and 2 mm thick PDMS layers were prepared as described above. These layers were then bonded using a 70 min UVO treatment. Each structure was prepared four times. Tubes of two different sizes (i.e., 1.0 and 1.6 mm outer diameters) were inserted, and the influence of insertion and slight and strong movement was investigated using a confocal microscope. Figure 2 provides a schematic overview of the structures, while Supplementary Materials Figure S4 shows the more detailed structure as used for preparing the microfluidic chip.

### 2.7. Static Analysis

A static analysis was performed using Autodesk Inventor Professional 2025 to investigate how the predetermined breaking structures affect stress propagation within the material. The material parameters summarized in Table 1 were used for the simulation of PDMS. There is little information on the parameters for UVO-treated PDMS surfaces, likely due to difficulties measuring thin layers. Song et al. [51] investigated the elastic modulus and reported values of 110 MPa on the surface and 37 MPa within the µm range after a 60 min UVO treatment. Since we do not mean to speculate on the other material parameters, we decided to only change the elastic modulus of the UVO-treated PDMS to 50 MPa as an approximation.

We tried different combinations to create thin and thick PDMS layers, working with several local meshes. However, in order to achieve a good simulation of the structure, the mesh should be optimized over a small number of iterations. This always led to the problem of the iterative process stopping due to an error caused by the different mesh sizes. Therefore, we divided the task into two simulations. First, we examined the impact of a change in elasticity on the bulk material surrounding the inlets, both with and without a predetermined breaking structure. Second, we combined thick PDMS layers with different elasticities to visualize the influence that the thin hardened layer would have on the PDMS in the first simulation.

For the first simulation, the structure shown in Figure S3 was split in the middle of the 1 mm inlets, and the portion containing the 1.5 mm inlets was considered transparent. The structure is 80 µm thick, and the total PDMS layer is 2.08 mm. For the analysis, the side and bottom areas were considered fixed, while the cross-sectional area is limited by the transparent second half of the PDMS body. The general mesh size was 0.05 mm, and two local meshes of 0.03 and 0.02 mm, respectively, were used for the area of the inlets and the edges of the structures. A force of either 2 N or 35 N was applied to the inlet area. In pure PDMS, 2 N increases the top diameter of the inlet from 1 mm to 1.06 mm, a realistic size for inserting a tube. However, in UVO-treated PDMS, 35 N is necessary for the same increase in diameter.

For the second test, we used a simplified inlet model with an inlet measuring 1 mm in diameter in a 2 mm thick PDMS layer. This layer was attached to either a 2 mm thick PDMS layer or four 0.25 mm thick UVO-treated PDMS layers. In the first case, we used a 50 MPa elastic modulus for the 1 mm layer. For the second case, we lowered the elastic modulus from 5 to 10 to 30 to 50 MPa for the different layers. A 2 N force was applied to the 2 mm layer. A 0.05 mm mesh was used, as well as a 0.02 mm local mesh for the edges.

Figure S7 shows the bodies for both simulations with the used mesh. The number of iterations for mesh optimization was set to 5 for all simulations, and von Mises stress (equivalent tensile stress) was used for optimization. It was increased if necessary. The number of iterations ranged between 1 and 6.

### 2.8. Sealing of the Tubes

**Figure 3:**
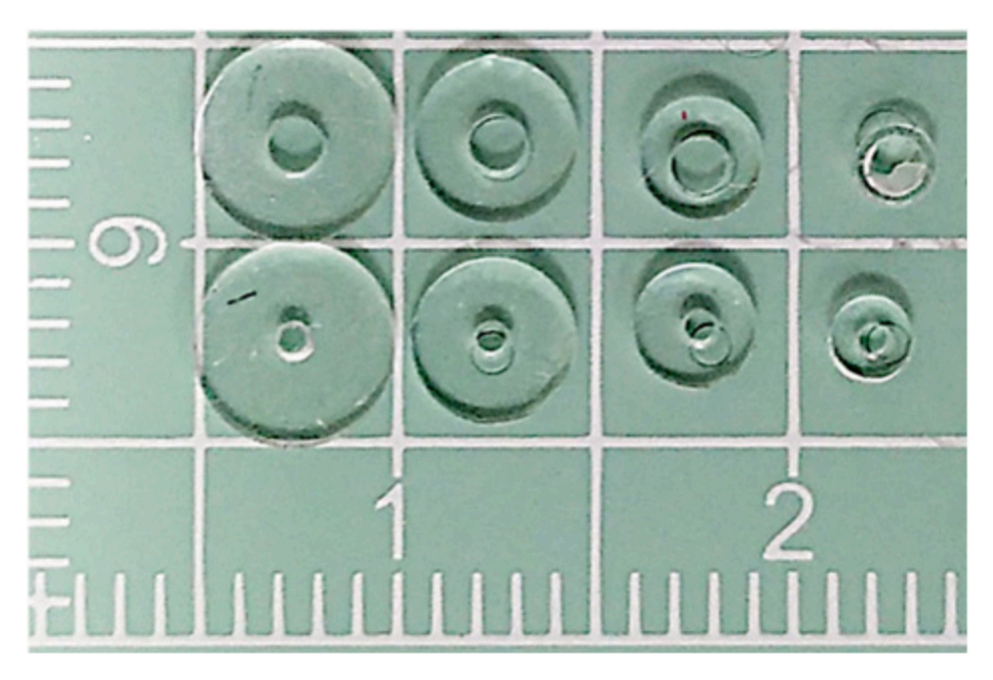
PDMS seals with 1.5 mm (top) and 1.0 mm inner diameter (bottom), 5, 4, 3 and 2 mm outer diameter (from left to right) and 0.5 mm thickness.

**Figure 4:**
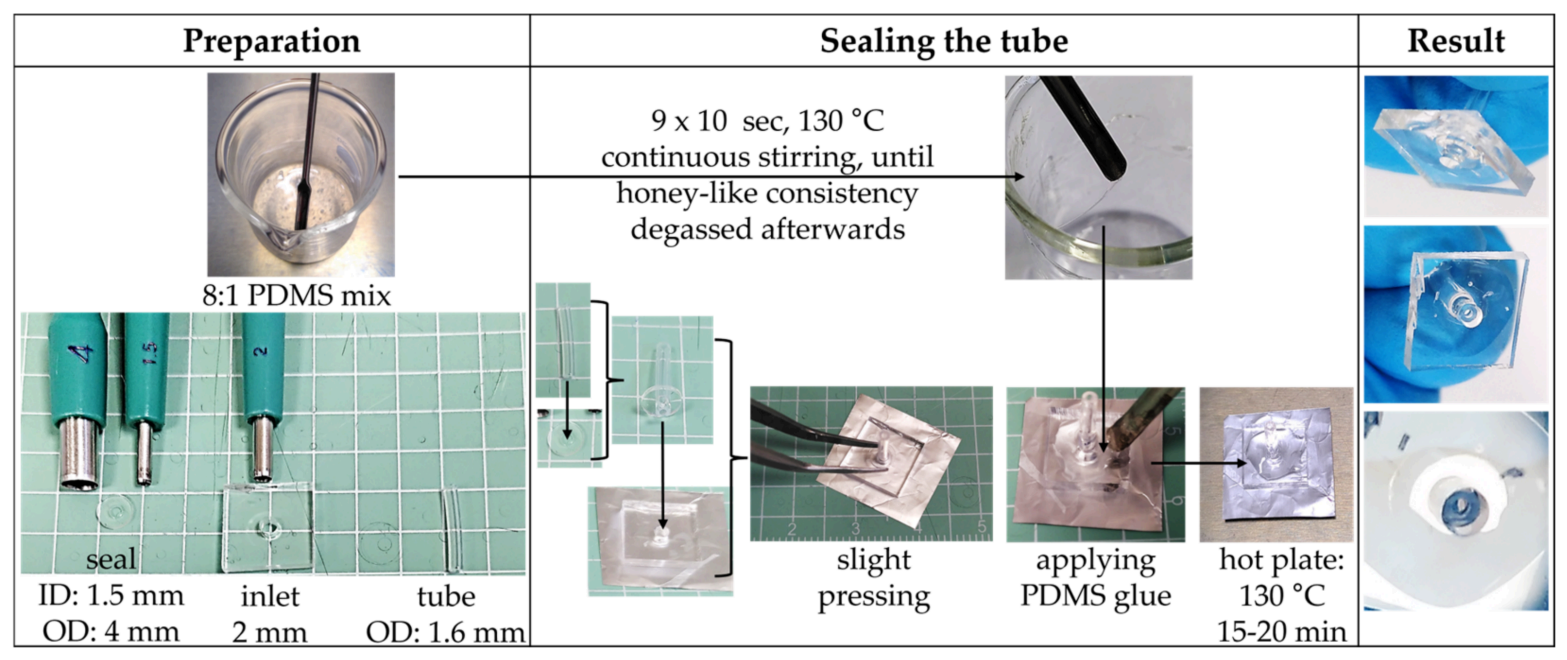
Overview of the procedure to seal a tube of 1.6 mm outer diameter (OD) to an inlet created with a 2 mm biopsy punch by using a seal with an inner diameter (ID) of 1.5 mm and OD of 4 mm.

If the PDMS does not seal the tubes automatically, for example, if the hole is too big or the walls are no longer flexible enough, the tubes must be sealed properly to avoid leakage. This was achieved by gluing the tubes with PDMS and curing them at a high temperature. However, applying freshly prepared PDMS to the small gap between the tube and layer is risky, as the PDMS can easily enter the gap and clog the microfluidic channel underneath. To prevent this, PDMS seals with an inner diameter of 1 or 1.5 mm, an outer diameter of 2, 3, 4 and 5 mm and a thickness of 0.1, 0.2 or 0.5 mm were prepared (Figure 3). A 1 cm tube with an outer diameter of 1 or 1.6 mm was inserted in such a seal and placed in an inlet of 1.5 or 2 mm size, respectively. Due to adhesion, the seal was able to slightly stick to the PDMS surface. Pre-polymerized PDMS glue was prepared by stirring an 8:1 PDMS mixture on a hot plate at 130 °C 9 times for 10 s each, until the viscosity was doubled. The glue was applied to the seal and fully cured on the hot plate at 130 °C. Afterwards, it was checked to see if the glue had clogged the inlet or not. An overview of the process is given in Figure 4.

### 2.9. Air Burst Test for Simplified Bonding Samples

Air- or water-based burst tests are a practical approach to quantifying the bonding strength, which directly demonstrate the operating limitations of microfluidic chips. For this determination, 2 mm thick PDMS layers were prepared on a blank wafer and cut into 1.5 cm × 1.5 cm pieces. Half of them were punched in the middle, and they were cleaned as described previously. Five samples each were prepared by assembling a punched piece and a non-punched piece after a UVO treatment of 60, 70, 75, 80 or 90 min. The starting temperature for the treatment was 25–26 °C in all cases. Then, the samples were placed in an 80 °C oven for at least one hour. Tubes with an outer diameter of 1.6 mm, seals with an inner diameter of 1.5 mm and an outer diameter of 4 mm and flexible silicone tubes with an inner diameter of 1 mm and an outer diameter of 3 mm were assembled and glued to the bonded samples with pre-polymerized PDMS. Figure S9 in the Supplementary Materials provides an example of the sample assembly. The samples were connected to a stepper pump and a pressure sensor. The pump was operated to increase the air pressure until a sudden pressure drop was reached.

### 2.10. FTIR Measurements

A silicon wafer was cut into 1.5 mm × 1.5 mm substrates. The substrates were cleaned in acetone and DI water for 5 min each and dried under nitrogen. Then, they were dehydrated at 150 °C for 5 min. Approximately 300 µL of a 10:1 PDMS mixture was spin-coated onto each substrate (4000 rpm for 3 min) and cured at 130 °C for 5 min. The thickness was measured three times for four samples using a profilometer with 0.1 mg contact pressure and a tip of 12.5 μm radius. Samples were UVO treated for 30, 60, 75 and 90 min. In comparison, a silicon substrate without coating and an untreated sample were measured.

### 2.11. XPS Measurements

A 1 mm thick PDMS layer with a 10:1 mixing ration was prepared at 130 °C for 20 min. The layer was cut into 10 mm × 10 mm pieces. Three samples were activated by a 60, 75 and 90 min UVO treatment and used for the XPS measurement. Measurements were performed within 4 h after the treatment. In comparison, an untreated PDMS piece and a glass substrate were measured as well. Survey scans were performed with 3 iterations and a pass energy of 200 eV, while high-resolution scans were performed at 20 iterations and a pass energy of 50 eV. For the analysis of the XPS spectra, the software CasaXPS (version 2.3.25) was utilized. Here, the Shirley algorithm was used for estimating the background of each high-resolution spectrum. Compositional analysis was performed by numerical integration of the area between the background line and the corresponding high-resolution spectrum data considering the peak specific relative sensitivity factors. To all spectra, a charge correction was implemented, setting the lowest carbon peak to 284.8 eV.

### 2.12. Leak and Water Burst Tests for Microfluidic Chips

**Figure 5:**
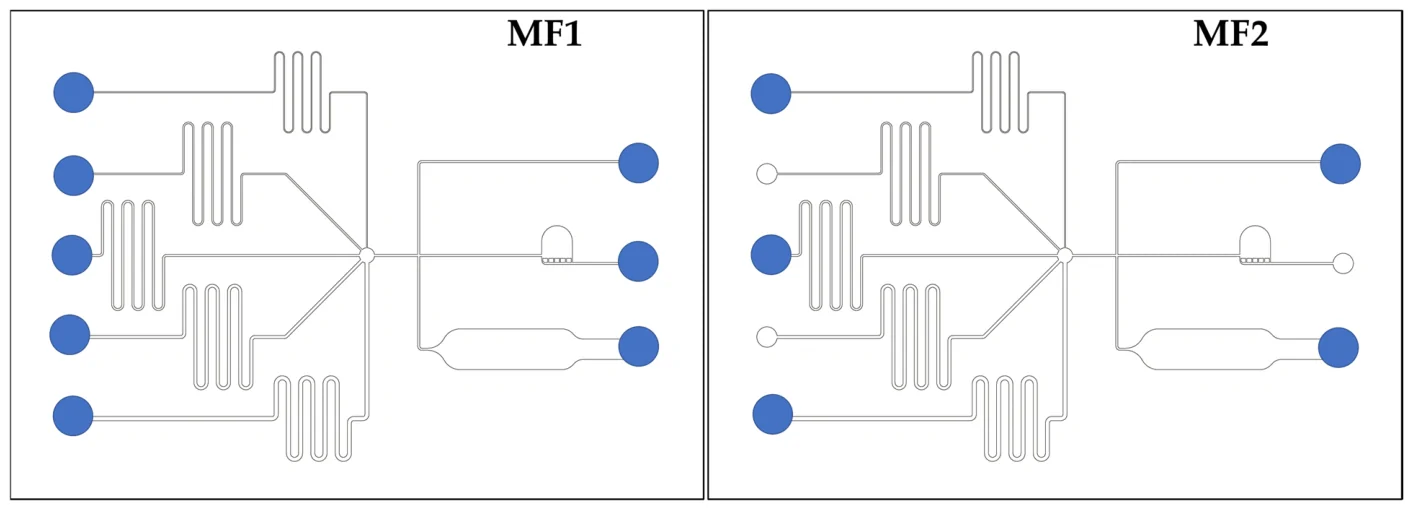
Structure of the two microfluidic chips (**MF1**) and (**MF2**) with the punched inlets marked with blue circles. The size of the chip is 24 × 34.5 mm. The structures contain channels with widths ranging from 50 to 200 µm and a length of 42.5 mm, as well as a simple outlet channel and a small or a large measurement chamber. The inlets used for preparing the microfluidic chips are marked in blue.

Six microfluidic chips were prepared to demonstrate that functional microfluidic chips are produceable using a UVO treatment. All PDMS layers were prepared using a 10:1 mixing ratio. A 2 mm thick unstructured PDMS substrate was used as the bottom layer. The structure of the top layer is shown in Figure 5. After punching, the layers were cleaned with acetone, dried at 130 °C and bonded following a 70 min UVO treatment. Afterwards, an at least 1 h post-treatment at 80 °C was performed. For MF1a–c, the marked inlets were punched with a 1.5 mm biopsy punch. Tubes with an outer diameter of 1 mm and PDMS seals with an outer diameter of 4 mm and an inner diameter of 1 mm were used. For MF2a–c, clogging of neighboring inlets might be a problem when gluing the tubes one after another. Therefore, only the two outer and the middle inlets were punched with a 2 mm biopsy punch. Tubes of 1.6 mm outer diameter and PDMS seals with an outer diameter of 4 mm and an inner diameter of 1.5 mm were used. The glue was made by stirring an 8:1 PDMS mixture on a hot plate at 10 s intervals until the viscosity doubled (in total 110 s). The tubes were glued while the chip was placed on a hot plate. Additionally, the glue was applied to the edge of the chips as protection during manual handling and to glue it to a glass substrate to prevent bending during the handling of the chip. The PDMS glue was cured on a hot plate at 130 °C for 20 min.

For the leak and burst tests, the inlets of the microfluidic chips were connected via Y-connectors and silicone tubes to a vessel containing blue-ink-colored water. The outlets were connected via Y-connectors to the tube with a pressure sensor and stepper pump. The pump’s flow rate was increased step by step, with the pumping direction always being changed to check the operation in a pressure- and vacuum-driven system within the same test. At the end of the test, all tubes were closed, while the pump ran to increase the pressure until leakage occurred. For MF1b, MF1c, MF2b and MF2c, the test was automated, and each pumping step was performed for 3 min. For the burst test, the flow was set to 170 µL/min, and all tubes were closed to increase the pressure until a sudden pressure drop of 200 mbar occured.

## 3. Results and Discussion

### 3.1. Optimization of the Individual Steps for the Microfluidic Chip Preparation

**Figure 6:**
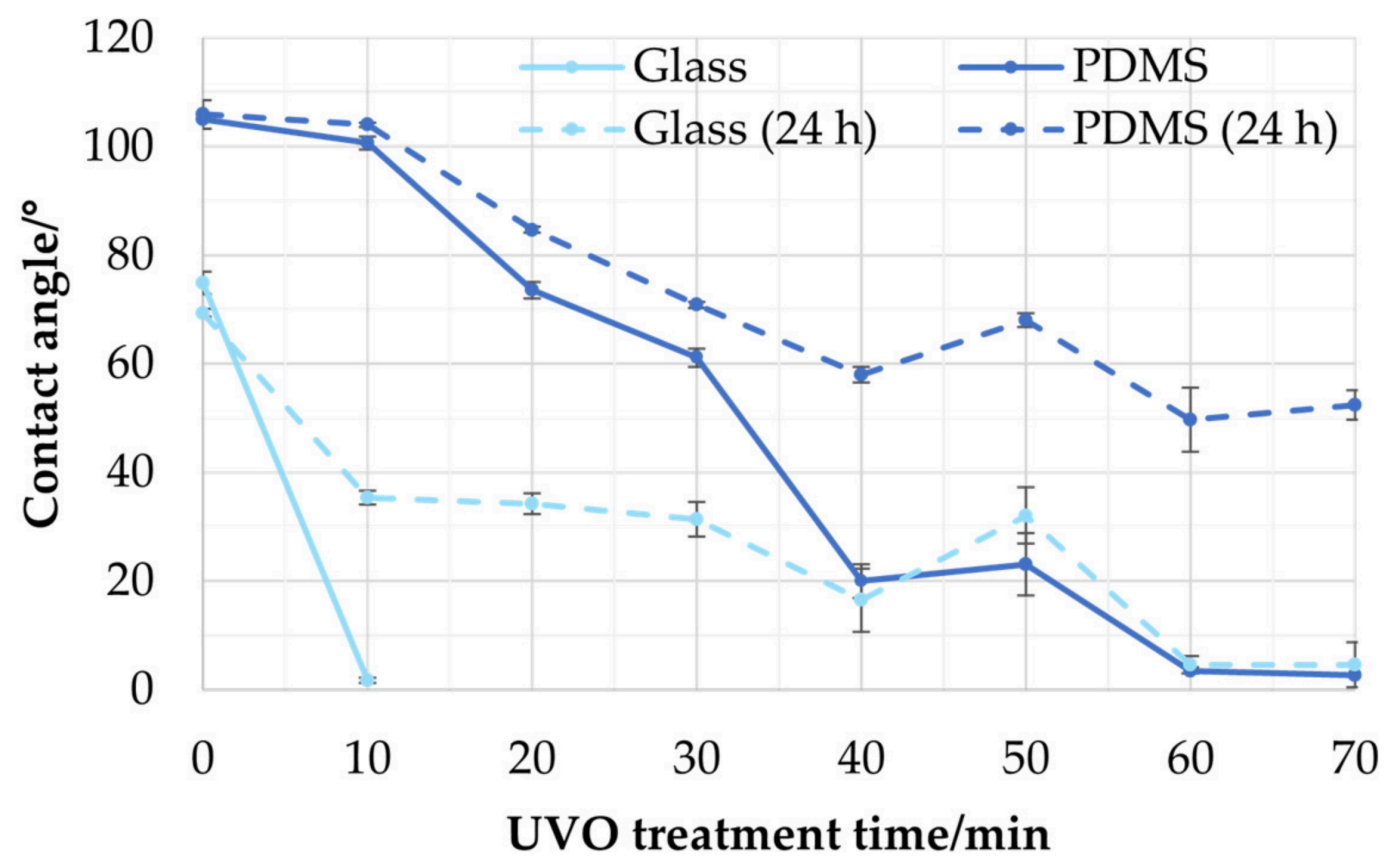
Results of the contact angle measurements of glass and PDMS depend on the UVO treatment time. The same samples were measured within a few minutes and 24 h after activation (dashed lines). Samples treated for 10, 30, 50 or 70 min and samples treated for 20, 40 or 60 min were analyzed together at starting temperatures of 20 °C and 30 °C, respectively. Figure S2 shows exemplary photographs of the droplets on the glass and PDMS surface a few minutes after the treatment.

Figure 6 shows the results of the contact angle measurements. After 10 min of UVO treatment, the contact angle of the glass decreases to an unmeasurable value. For PDMS, the contact angle steadily decreases and reaches an average value of 2.6° after 70 min. The comparatively lower values of the samples treated for 20, 40 and 60 min are due to the fact that these samples were activated at an initial instrument temperature of ~30 °C, whereas the initial temperature for the other samples was ~20 °C. A higher temperature leads to faster activation. In both cases, the samples become less hydrophilic over time. This finding is consistent with other studies. Due to UVO treatment, the surface structure of PDMS is partially converted and permanently altered. In comparison, the PDMS activation with an oxygen plasma etcher usually takes only a few min, and the hydrophobicity fully recovers over a longer period of time because the treatment only changes the surface, not the bulk material [30,31].

The peel test results for all tested conditions are given in Table 2 and Table 3, which show the results for PDMS@glass and PDMS@PDMS bonding, respectively. In the peel test, the samples were categorized as follows:

- No bonding (colorless).
- Less than half of the area was bonded, and/or the two layers could be separated with minimal force (yellow).
- More than half of the area was bonded, and the two layers could only be separated by destroying the PDMS (blue).
- The whole area was bonded, and the two layers could only be separated by destroying the PDMS (green).

Although bonding was exceptionally possible, no reproducible working conditions for bonding glass and PDMS were identified within the range of 10–90 min PDMS activation and 20–90 min glass substrate activation. However, bonding was possible in some cases, and the hydrophilization of the glass was confirmed by the contact angle measurements. Therefore, it is presumed for the problem that the lower flexibility of the PDMS surface does not allow the materials to connect well enough for the formation of chemical bonds across the entire surface. When taking into account the possibility of PDMS@glass bonding described in the literature, this suggests that the surface hardening might be less prominent with other instruments, This could be due to a different temperature, lamp intensity, or distance between the glass and the UV lamp. The latter is variable in some available UVO instruments and influences the intensity. However, our instrument does not allow these parameters to be modified precisely.

However, 70 min of treatment is sufficient for PDMS@PDMS bonding, and PDMS does not become damaged when left to activate for longer (i.e., for 80 or 90 min). For the samples marked in blue in the 70 min test, only small spots on the edges of the PDMS pieces did not bond. Therefore, this time is useable to prepare microfluidic chips with a distance of 2–3 mm between structures and the edge.

Additionally, 110 µm thick PDMS membranes were tested. However, weak bonding was observed in several tests when activation times of 10, 15 or 20 min were used. After 20 min of UVO treatment, the bonding was strong enough to prevent delamination when tubes were inserted in only one out of four cases. Beyond 20 min of UVO treatment time, the surface became rough, repellent and slightly opalescent. Moreover, it was observed that this surface change became more pronounced at higher temperatures within the range of 20–32 °C, as the instrument heated up during use within that range. This suggests that activation at temperatures below 20 °C might produce more consistent results, but this was not investigated in the context of this study. However, when PDMS is spin-coated onto a glass substrate and cured to form a layer of the same thickness as the membranes, it was successfully activated for 70 min and used in PDMS@PDMS bonding. Berdichevsky et al. [33] described the formation of cracks at high temperatures and ascribed it to the hardened surface and an up to 30% shrinkage level of the treated layer. This shrinkage value is most likely different in our case, as it depends on the lamp intensity, which is not given. However, this suggests that the combination of the membrane’s high flexibility and its hardening due to ozone exposure results in micro-cracks forming on the surface. The increase in surface roughness could then cause the weak bonding.

Furthermore, reverse bonding of the membrane was tested as a workaround. For this, a spin-coated glass substrate was bonded to a PDMS microfluidic layer using the 70 min ozone treatment. The 0.11 mm thick membrane was then carefully peeled off the glass substrate. It was damaged when reaching a 1.5 mm diameter cut-out in the PDMS layer, most likely due to a combination of high mechanical stress and the slight hardening of the membrane. For this study, we concluded that microfluidic chips should be prepared using spin-coated glass or PDMS substrates, and PDMS membranes should be avoided.

Punctual pressure comes with a risk of crack formation in the surface, and the PDMS@PDMS bonding strength is also weaker in comparison to activation with the oxygen plasma treatment [33,34]. This was observable when we performed the first tests to prepare microfluidic chips with the 70 min UVO treatment time (see Supplementary Materials Figure S3). In order to minimize the impact of these cracks and weak bonding, microfluidic chips with the predetermined breaking structures surrounding the inlet were prepared using photolithography and soft lithography processes. Cracks still appeared in the bonded surfaces when a 1 mm or 1.6 mm tube was inserted into an inlet created with a 1 mm or 1.5 mm biopsy punch, respectively (see Supplementary Materials Figure S5). This is because the PDMS bends during punching, creating a hole with a smaller diameter than that of the puncher used. When the tube is inserted, the resulting strain within the PDMS leads to cracks in the surface.

**Figure 7:**
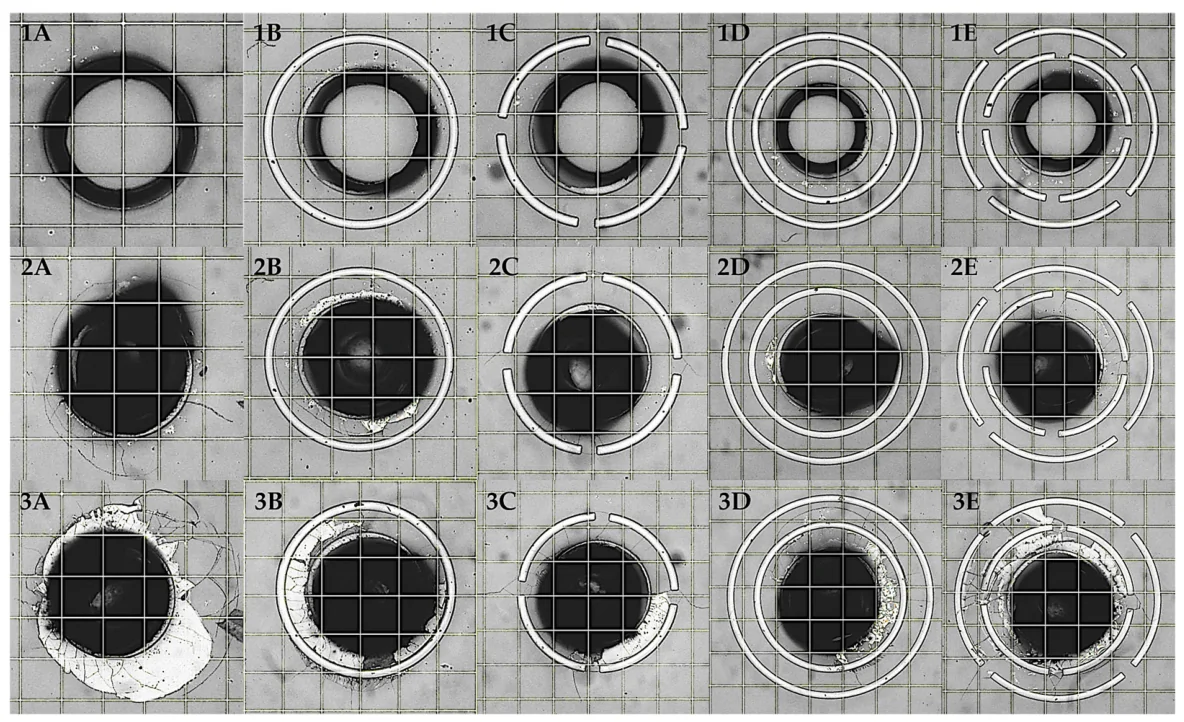
Micrographs of 1.5 mm inlets with different predetermined breaking structures (**A**–**E**) before inserting a tube (**1**) and after inserting 1 mm tubes and moving them to mimic usual handling of a microfluidic device (**2**), as well as after moving and pressing with extended force to purposefully damage the structures (**3**). The test was performed with four different chips, and the presented pictures are examples. All pictures are given in Supplementary Materials Table S1. For all pictures, the contrast and sharpness were increased for better visualization of the damages. A square equals 500 µm × 500 µm.

Further mechanical stability tests on the structures were therefore performed by adding a 1 mm tube to a 1.5 mm inlet. Depending on how much the PDMS bends during punching, this either fits exactly or is slightly too small, leaving a small gap between tube and PDMS wall. Each structure was prepared four times. Examples of the micrographs are given in Figure 7, and all pictures are compiled in Supplementary Materials Table S2. The tubes were moved and pressed to mimic the careful handling of a microfluidic chip during laboratory testing. As observable in Figure 7(2A–2E), this results in small cracks or punctual breaking of the bonding (as indicated by the lighter-colored spots), but the structures could still be used even without the predetermined breaking cycles.

However, long-term use or careless handling results in the elongation of the cracks. Therefore, the PDMS around the tubes was deliberately damaged by pressing and moving them with greater force in order to identify their weak spots. Some of the resulting images are shown in Figure 7(3A–3E). The lighter areas are due to a broken bond and a detachment of the PDMS layer.

**Figure 8:**
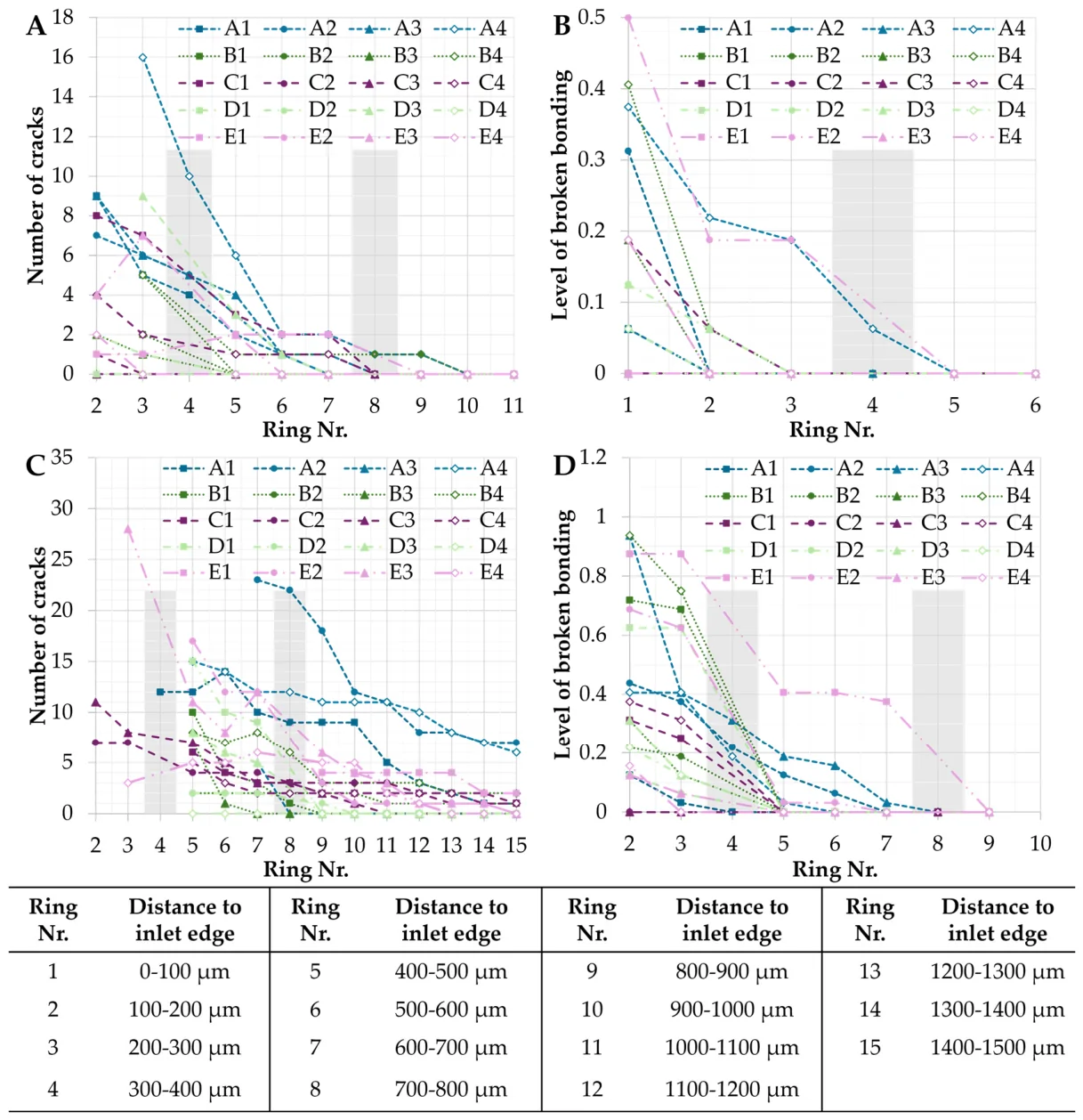
Evaluation of the number of cracks (**A**,**C**) and the level of broken bonding (in the range 0–1, with 1 = total area detached; (**B**,**D**) for the 4 tests of inlets A–E after simulation of usual handling of a microfluidic chip (**A**,**B**) and forceful handling of a chip (**C**,**D**). The area of the predetermined breaking structure is marked in gray. The counting of cracks was started when no visible detachment was present in a ring area.

**Figure 9:**
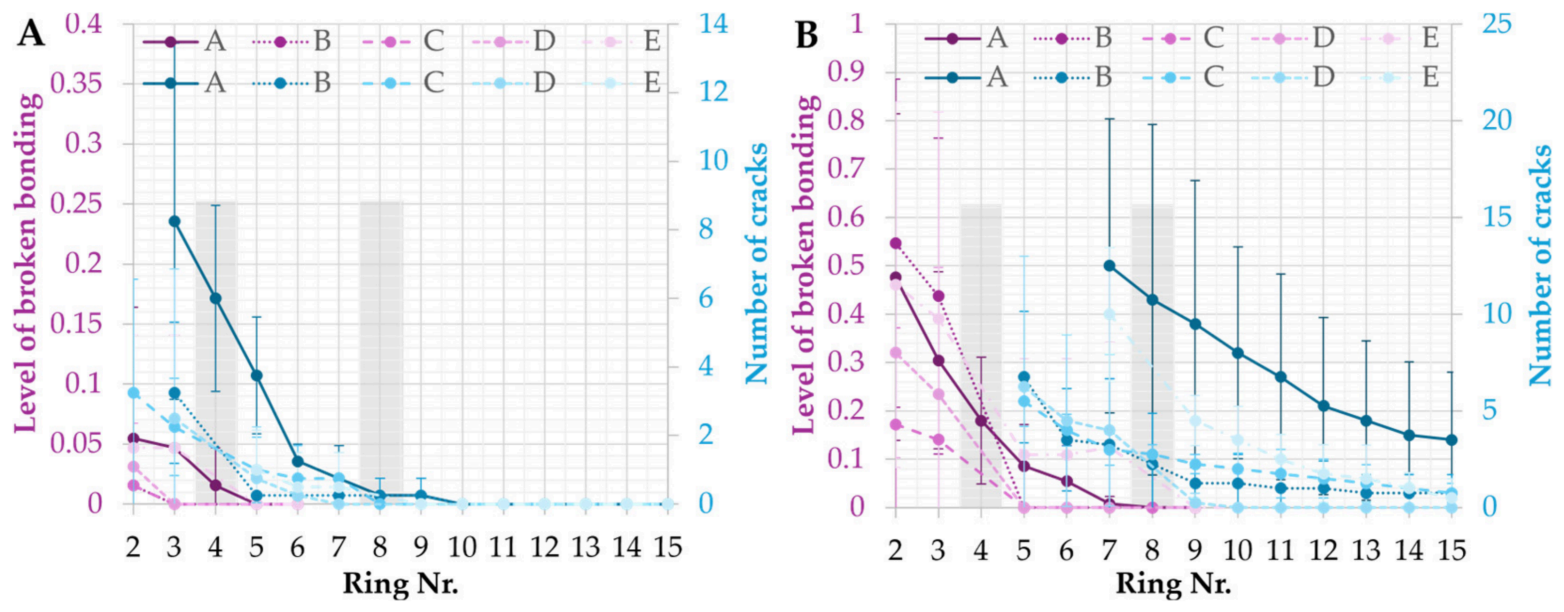
Average level of broken bonding (pink) and the number of cracks (blue) in the different ring areas surrounding inlets A–E of the four tested samples. Graphs (**A**,**B**) illustrate the average results of the usual and careless handling tests, respectively. The area of the predetermined breaking structure is marked in gray. The counting of cracks was started when no visible detachment was present in a ring area.

To visualize general trends, the area surrounding the inlets was divided into 15 rings, each 100 µm wide, separated by 16 lines, each rotated 22.5°. Figure S6 provides an example. This method was used to determine the amount of detached area due to broken bonding and the number of cracks within each ring within the range of 100–1500 µm from the edge of the inlet. The first ring was not taken into account, since the shadow of the tube often covered this area partially. The area in which the bonding is broken is determined by a value between 0 (no detachment) and 1 (complete detachment). Figure 8 and Figure 9 show the individual and the average results of this evaluation, respectively.

The carefully handled samples show that the inlets with predetermined breaking structures tend to have fewer and shorter cracks when the amount and length of the cracks are determined (Figure 9A). However, one sample from inlet A had much higher values (Figure 8A), which resulted in higher values and a higher standard deviation, and therefore, the impact of the predetermined breaking structures seems to be small. In all cases, areas more than 1 mm away from the inlet are not harmed. Therefore, these structures are not necessary if the chips are handled carefully and the distances between the inlets are chosen accordingly. If the force is increased to cause further damage to the inlet, the number of cracks in inlet A will increase (Figure 9B), i.e., there will be more long cracks, which pose a higher risk of long-term damage to the microfluidic chip. The area of broken bonding for inlets B, C and D is similar to that of inlet A. However, it drops to almost 0 due to the first ring in the predetermined breaking structure. This is not the case for one of the inlets, E, which was much more damaged (Figure 8D). This leads to higher values and standard deviations. However, the predetermined breaking structures reduce the risk of long cracks, though they cannot prevent them completely. In some cases, one or two long cracks remain, especially at the interrupted ring area of inlets C and E. This area tends to elongate the cracks. Therefore, the uniform rings around the inlets and outlets are a better choice. Two ring structures need more space, so depending on the microfluidic device, one ring structure might be more sufficient in some cases. This might extend the lifetime of the microfluidic chip during handling in a laboratory setting, for example, when tubes are repeatedly connected to other devices or when a chip is accidentally dropped.

The static simulation visualizes the impact of applying a force—in our case, inserting a tube—on the material and structure. The most important parameters are von Mises stress, which combines tensile, compressive and shear stresses, and the safety factor, which indicates the distance to the load limit of the material. If the safety factor falls below 1, it indicates permanent damage to the material. However, we used a linear elastic model for a hyperelastic material. Therefore, the model is an approximation, not an exact representation. Although we can use it to observe general trends by directly comparing structures exposed to the same influencing factors, we cannot rely on precise values or direct comparability with actual measurements, which are therefore not discussed below.

**Figure 10:**
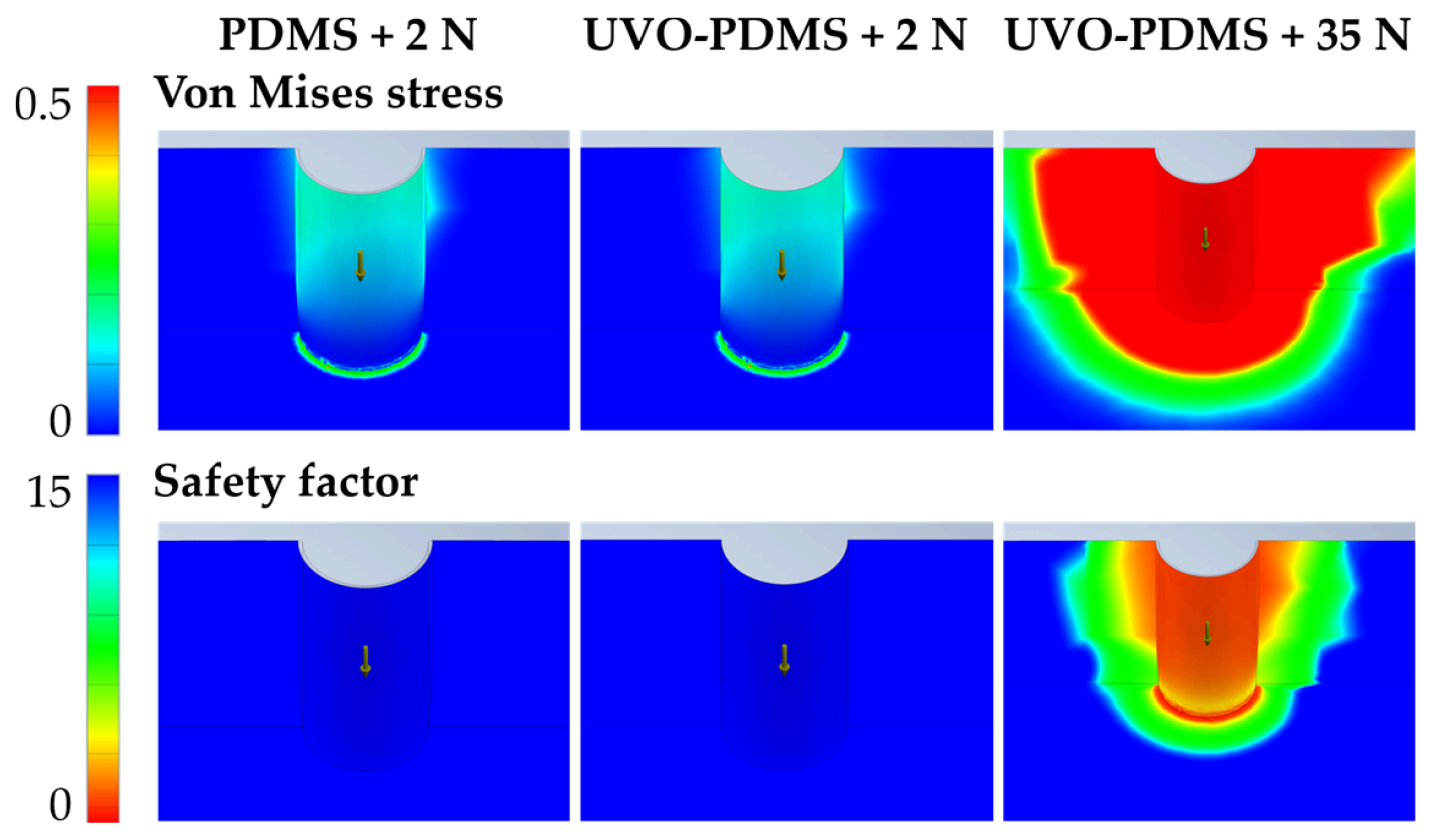
Simulation of von Mises stress and safety factors for inlet A in PDMS and UVO-treated PDMS when a force of 2 or 35 N is applied. The arrow indicates the force applied to the area of the inlet. The simulation results on the left show how PDMS would deform if a force of 2 N were applied, using the values from Table 1. The results in the middle show what happens when a force of 2 N is applied to UVO-treated PDMS with a higher elastic modulus. On the right, the force was increased to 35 N to achieve the same level of deformation in UVO-treated PDMS as in the result on the left.

Figure 10 shows the von Mieses stress and the safety factor for the inlet without predetermined breaking structures when the material changes and the force is increased. A 2 N force leads to a widening of the inlet from 1 to 1.06 mm. For PDMS chips bonded with a plasma etcher, this widening of the inlet by a tube is easily compensated by the elastic material, without risking damage of the chip. The same force leads to less bending of the UVO-treated PDMS. Meanwhile, the von Mises stress and safety factor are similar to those of the untreated PDMS. However, increasing the force to 35 N to achieve a similar degree of inlet widening, which, for both materials, would be necessary when inserting the same tubes into a microfluidic chip, drastically lowers the safety factor. This comes with the risk of damaging the material, as observed for the tested inlets.

**Figure 11:**
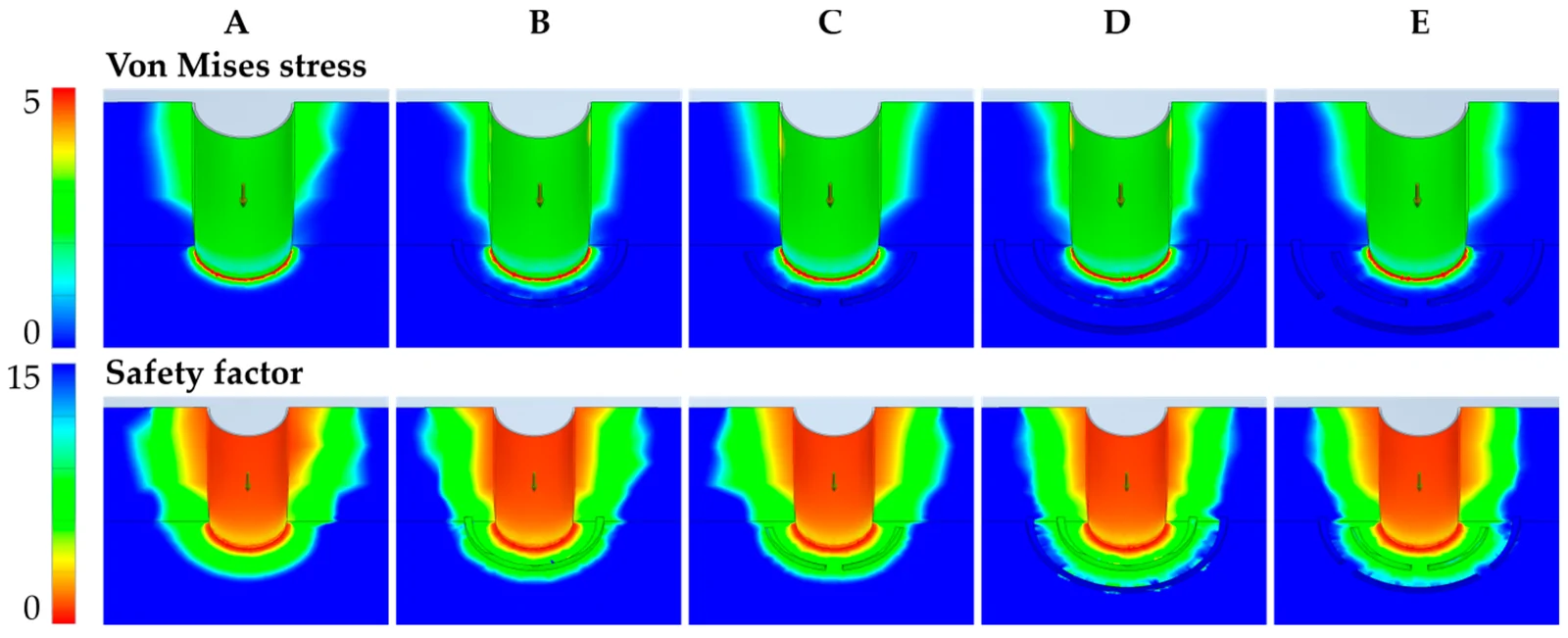
Von Mises stress and safety factor of different inlet structures (**A**–**E**) in UVO-treated PDMS when 35 N is applied. The arrow indicates the force applied to the area of the inlet.

Figure 11 illustrates how the von Mises stress and safety factor vary for different ring structures when 35 N is applied to UVO-treated PDMS. The von Mises stress is nearly identical, with most of the stress occurring at the bonded edge of the inlet. The safety factor is lowest at the edge of the inlet. It increases in the area of the first and second ring structures due to the greater distance from the bending material.

**Figure 12:**
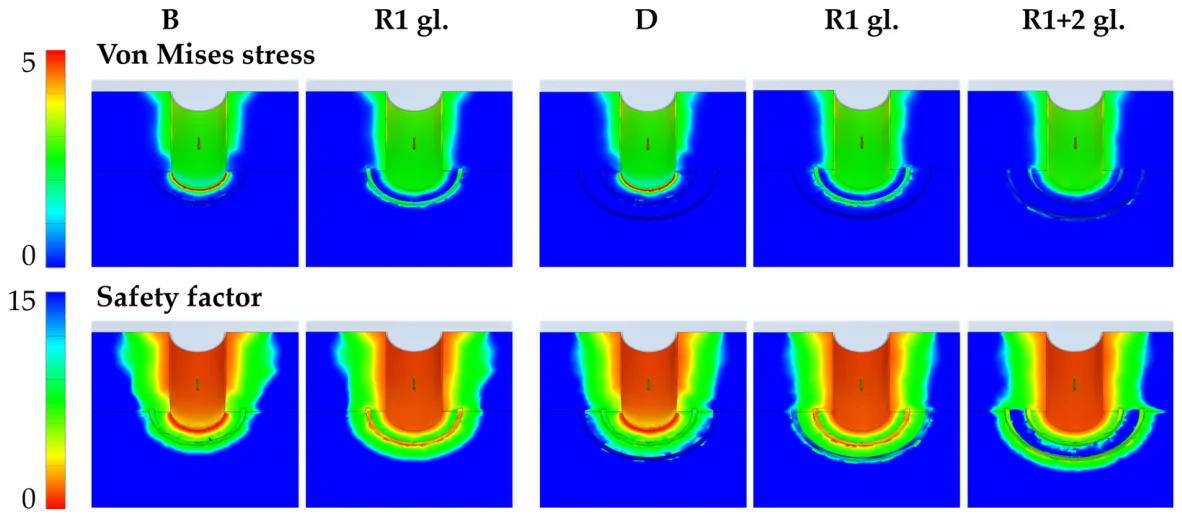
Von Mises stress and safety factor of inlet (**B**,**D**) if the first ring (**R1**) or both rings (**R1+2**) are allowed to glide, mimicking broken bonding. The arrow indicates the force applied to the area of the inlet.

However, if the bond breaks, the force will be distributed differently. To model this, we ran another simulation in which the first and second rings glide on the surface, mimicking a broken bond (Figure 12). This leads to a partial stress propagation to the material surrounding the ring, which is especially noticeable when the first ring becomes loose. This effect is less prominent for the second ring, presumably due to the structure’s higher overall flexibility. With that, the safety factor is lowered around the ring structures. However, the safety factor is still higher than at the edge of the inlet; therefore, the risk of damaging the material surrounding the predetermined breaking structure is reduced.

**Figure 13:**
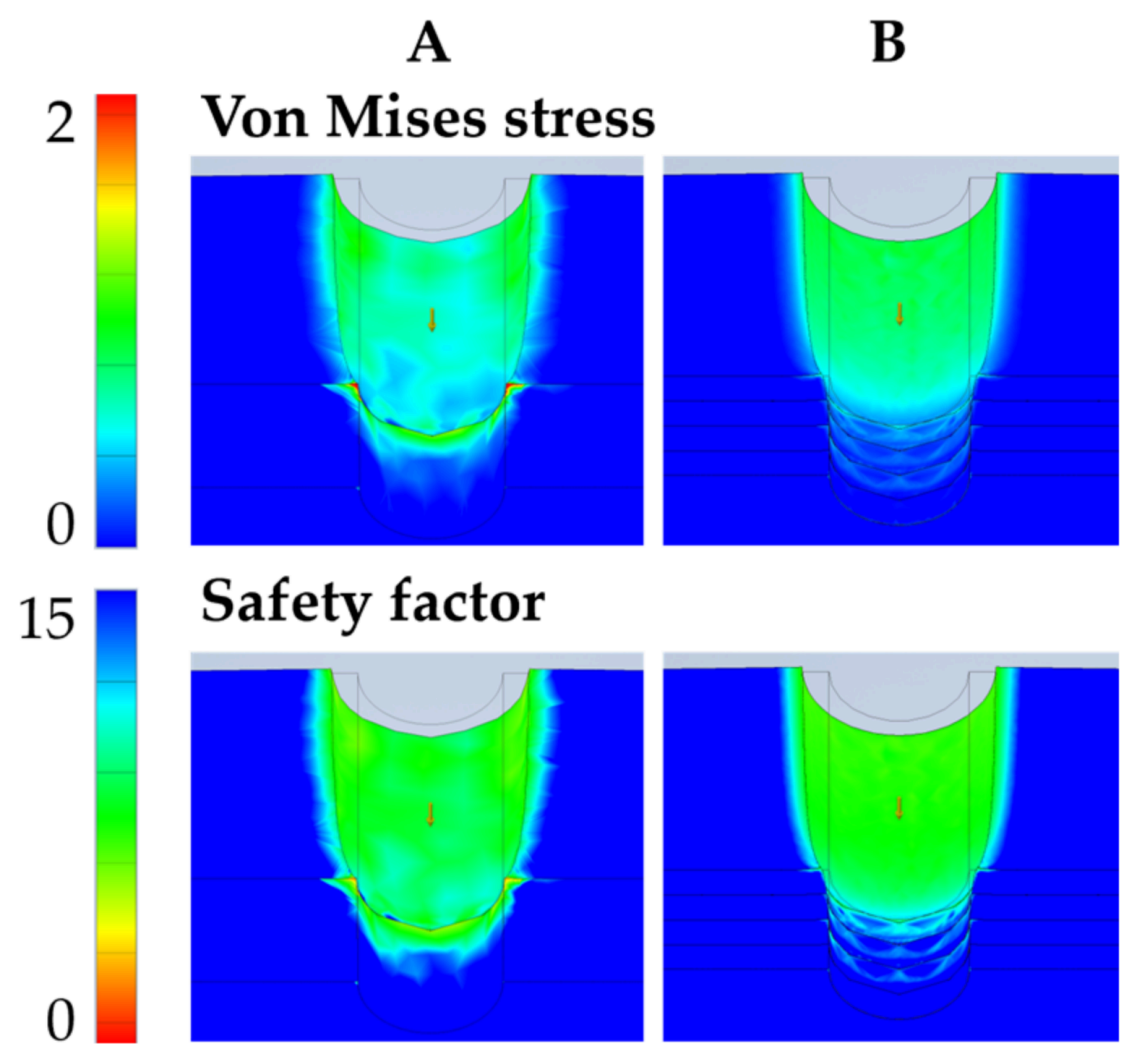
Von Mises stress and safety factor for the simplified inlet model. In case (**A**), the elastic modulus was set to 2.5 (2 mm layer) and 50 MPa (1 mm layer), and in case (**B**), the 1 mm layer was split into four 0.25 mm layers with elastic moduli of 5, 10, 30 and 50 MPa (increase in surface direction). The arrow indicates the force applied to the area of the inlet.

The simulation of the simplified inlet model shows a damping effect due to the different elastic moduli of the four layers for both the von Mises stress and the safety factor (Figure 13) when a force is applied from the PDMS site. In reality, the change in elasticity would gradually occur within the micrometer or nanometer range from the surface rather than forming a layered structure. This would lead to smoother damping. However, if a force is applied to the UVO-treated PDMS, the propagation would be reduced due to the lower elasticity, resulting in more prominent stress within the hardened surface layer and less stress in the bulk material. Therefore, the bulk effect in the first simulation set is presumably smaller than shown in Figure 10, Figure 11 and Figure 12.

In addition to the linear elastic model, there are other factors influencing stress and the risk of breakage that cannot be properly accounted for in these simulations. UVO treatment of PDMS results in a density loss of up to 30% [33]. Therefore, it is very likely that it comes with residual tensile stress within the amorphous molecular network. Moreover, the force was evenly distributed across the chosen surface. When handling microfluidic chips manually, the force is sometimes directed and spikes, for example, during the assembly. In the very thin surface layer, punctual stress cannot be distributed well because the underlying material is untreated and very flexible. This leads to the cracks we observed when inserting tubes of the same size or larger than the inlet, and it is necessary to use tubes smaller than the punched inlet to avoid this.

The main problem when using biopsy punches that are larger than the tubes is ensuring that the inlets and outlets are properly sealed without blocking the channels when the tubes are glued with pre-polymerized PDMS. As a solution, different PDMS seals were prepared. The seals with 2 mm outer diameter were not tested further, as they were difficult to handle and easily damaged. Moreover, the 0.1 mm and 0.2 mm thick seals were difficult to handle as they bent easily, allowing glue to enter the space between the tube and the wall. Additionally, when an outer diameter of 5 mm was used, the tubes could easily be pulled out after the glue had cured, resulting in a lack of mechanical stability. For the 1 mm and 1.5 mm tubes, seals of 3–4 mm outer diameter and 0.5 mm thickness were the easiest to use. In some cases, the seals did not fully prevent the PDMS from entering the space between the tube and wall, but it was getting in so slowly that the channels were not clogged, as observable in Supplementary Materials Figure S8. We found that using pre-polymerized PDMS glue to seal microfluidic chips is helpful when bonding or leakage problems occur. The viscosity of freshly prepared PDMS is usually too low to prevent it from entering very small gaps before it is fully cured. With pre-polymerized PDMS, however, this risk is much lower, and the glue is applied easily with a spatula when it has a honey-like consistency. Using a stand to stabilize the tube positions while gluing, with the chip already placed on hot plates, would greatly improve this process. However, compared to oxygen plasma-treated samples, where the punched holes can be smaller than the tubes’ outer diameter and the PDMS seals the tubes without glue, this process is more susceptible to errors.

To investigate bonding and gluing stability, simplified samples with just one inlet and one connected tube were prepared. These samples were connected to a DC pump and a pressure sensor, and a burst test was performed using air as the medium. Table 4 shows the maximum pressure observed during the test, and Figure S10 shows the measurements. However, since the pressure sensor does not provide accurate readings above 1000 mbar and should not be operated far below this upper limit, all samples with maximum values above 1000 mbar were retested at a pressure of approximately 1000 mbar for 10 min each (Figure S11). The results indicate that the highest bonding strength is achieved after a UVO treatment time of 75 min, likely due to a trade-off between surface activation and hardening. About half of the samples treated above 1000 mbar leaked due to glue detachment. Therefore, the PDMS glue can only be used up to a pressure of 1000 mbar.

**Figure 14:**
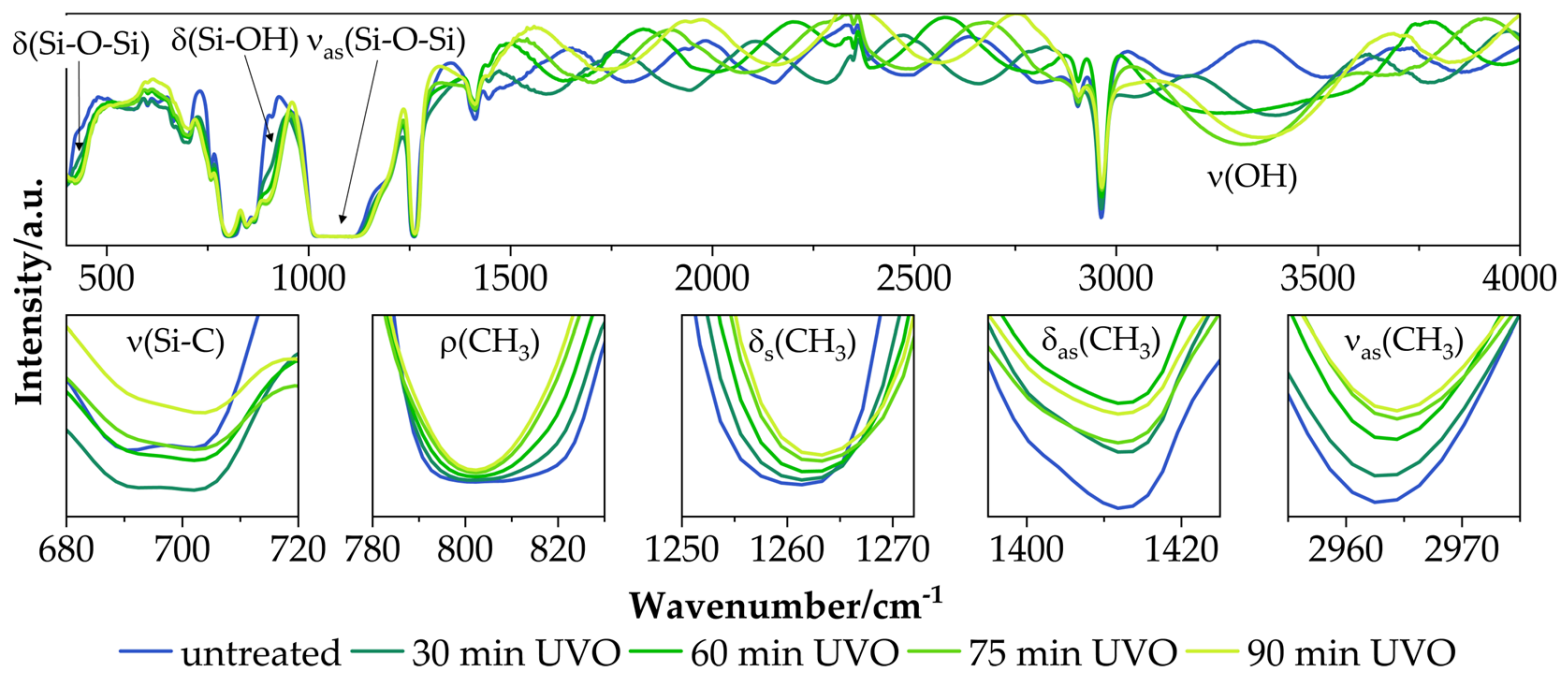
FTIR spectra of PDMS@Si samples after 30, 60, 75 and 90 min UVO treatment time in comparison to an untreated sample. A measurement of blank Si substrate was used as the reference.

Figure 14 provides an overview of the measured IR spectra, as well as cut-out sections of some significant peaks for 8.98 ± 0.13 μm thick PDMS layers after 30, 60, 75 and 90 min UVO treatments in comparison to untreated PDMS. Due to the etalon effect, a wave function occurs in the background. Several correction options were tested but produced less comparable results than the raw data, most likely due to small variations in the layer thickness and overlapping with the peaks, especially the broad ν_as_(Si-O-Si) and the ν(OH) peaks. This results in less comparability of the signals with low intensity, due to changes in intensity and shifting peaks, as can be seen with the ν(OH), ν(Si-C) and δ_as_(CH_3_) peaks. Therefore, a precise quantitative analysis of the signals is not possible. However, some general trends can be observed. The intensities of the ρ(CH_3_) (802 cm^−1^), δ_s_(CH_3_) (1262 cm^−1^) and ν_as_(CH_3_) (2962 cm^−1^) peaks decrease, while the intensities of the δ(Si-O-Si) (426 cm^−1^), δ(Si-OH) (895 cm^−1^) and ν(OH) (3000–3500 cm^−1^) peaks increase. This confirms the oxidization of the methyl groups, as well as the surface activation and crosslinking within the material. This finding is consistent with other reports [32,33,54]. The spectra of the samples treated for 75 and 90 min are mostly similar. One possible explanation might be that the material close to the surface is fully converted after 75 min treatment time, so the resulting shell slows further oxidization of the bulk material.

**Figure 15:**
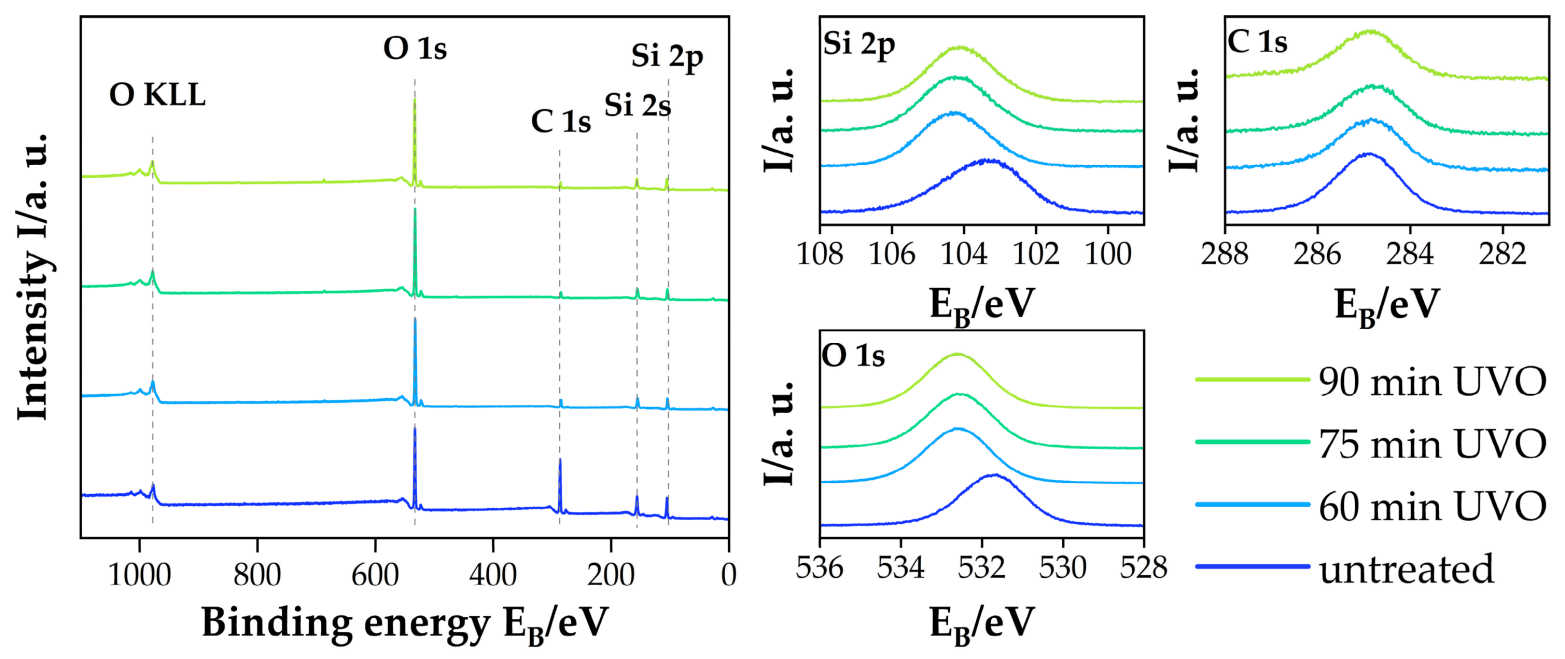
XPS survey scans (right) and high-resolution spectra of the Si 2p, C 1s and O 1s regions (left) of the 60 min, 75 min and 90 min UVO-treated PDMS samples compared to an untreated sample.

Table 5 summarizes the atom distribution as determined from the XPS overview scan shown in Figure 15. Untreated PDMS contains ~6% less carbon, ~3.1% more oxygen and 3.9% more silicon than theoretical values. This discrepancy may be due to several factors. Slight differences in the mixing ratio affect the atomic composition. Moreover Wen et al. [55] describe surface segregation of PDMS end groups and Si surface enrichment, which depends on the substrate and curing temperature, leading to an increase in the amount of silicon. Additionally, a decrease in carbon could be due to surface oxidation during storage in ambient air or the presence of silica filler in Sylgard 184 [56]. However, compared to untreated PDMS, a clear decrease in carbon and an increase in oxygen and silicon can be observed. The further increase in oxygen and decrease in carbon, depending on the treatment time, confirms that the conversion is ongoing, although it appears to slow down within this time range compared to the first 60 min. We observed 11% carbon on a glass substrate, likely due to adsorbed carbon impurities from storing the samples in Fluoroware^®^ boxes prior to XPS measurements. We calculated the theoretical formula sum for the 75 min treated sample, which shows an excess of silicon and carbon compared to the necessary oxygen for SiO_2_ formation. Therefore, it is likely that the oxidation process within the measurement depth range of up to 10 nm is not yet complete after 75 min.

Figure 15 (right) shows the stacked signals of silicon, oxygen and carbon of the PDMS samples. For the untreated sample, a comparison with the literature data was performed using 284.4 eV and 284.8 eV as the correcting reference for carbon, as in both cases, not all signals fit perfectly to the literature data. The summary is shown in Table 6. Using 284.4 eV as a reference, the silicon signal at 101.1 eV and the oxygen signal at 531.2 eV would be too small for PDMS. Using 284.8 eV as a reference, the carbon signal at 284.8 eV and 285.6 eV would not fit PDMS or other typical impurities very well. The oxygen peak at 531.6 eV also does not fit the reference perfectly. However, using the reference of 284.8 eV leads to better-fitting results for PDMS and is therefore used for all samples. These differences can be attributed to the Sylgard 184 kit, which contains, in addition to the dimethylated polymer chain, dimethylvinylated, trimethylated and trimethylsilylated silica; an inhibitor; and terminal dimethylvinylated polymer chains [57].

**Figure 16:**
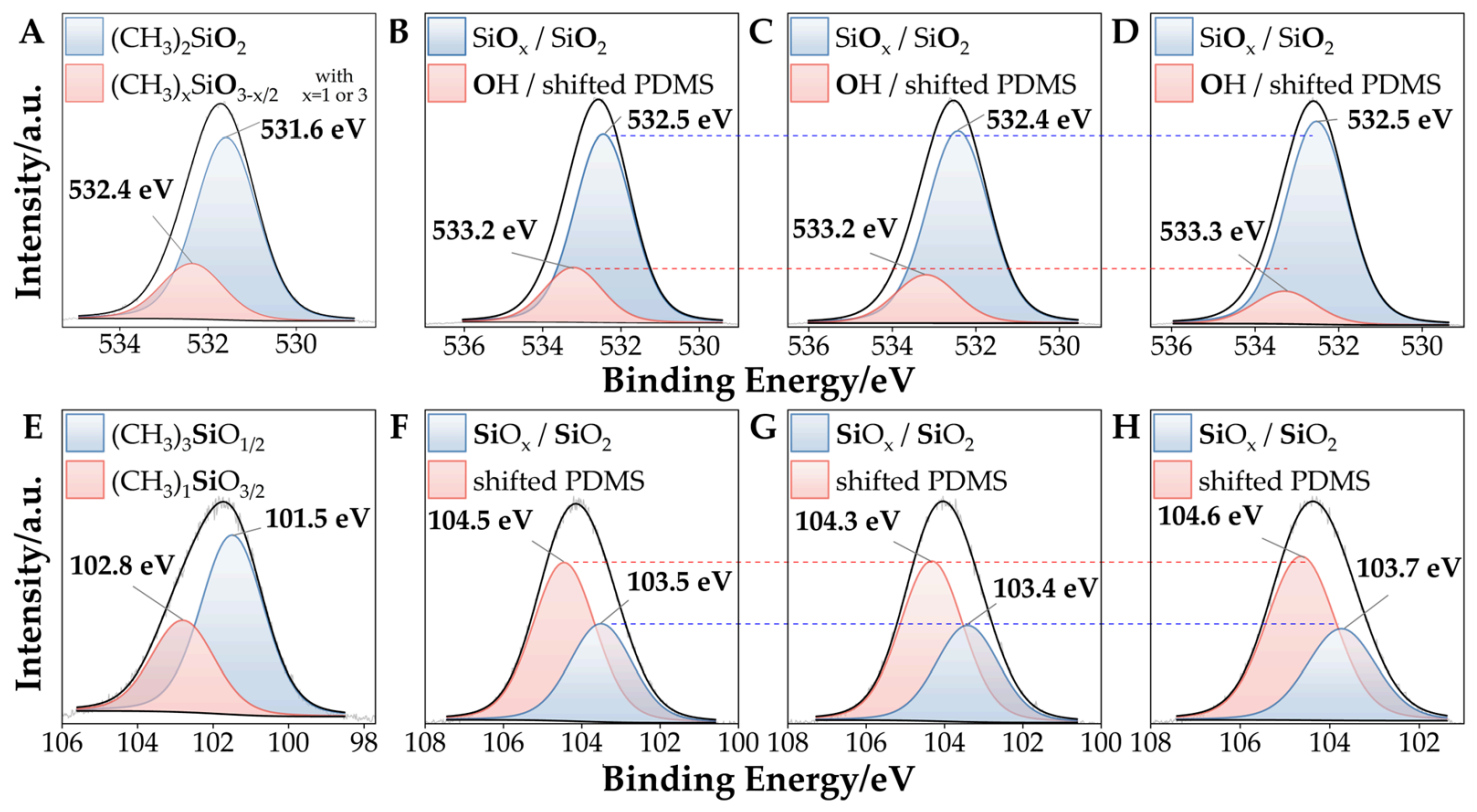
O 1s (**A**–**D**) and Si 2p (**E**–**H**) signals for the 60 min (**B**,**F**), 75 min (**C**,**G**) and 90 min (**D**,**H**) UVO-treated PDMS in comparison to the untreated PDMS (**A**,**F**).

Figure 16 shows the O 1s (A–D) and Si 2p (E–H) signals for the 60 (B,F), 75 (C,G) and 90 min (D,H) UVO-treated PDMS compared to the untreated PDMS (A,E). In all cases, the shift to higher values can be attributed to oxidization. However, in the case of the Si signal, the peak at 103.4–103.7 eV could be assigned to Si-O in SiO_x_ or SiO_2_ [59]. But the peak at 104.3–104.6 eV is higher than expected for the different compositions of partially oxidized PDMS and cannot be explained by chemical reasons but rather by a physical measurement artefact: Greczynski et al. [61] investigated the signal shift in PDMS samples of different thicknesses due to the charging, and Suzer et al. [62] described shifting signals of layered PDMS@SiO2@Si samples due to the charging. We are measuring a thin SiO_x_/SiO_2_ layer on a thick PDMS layer. Charging of the SiO_2_ surface and the partially oxidized PDMS therefore occurs differently and cannot be fully compensated. We believe this results in a shift of the signals towards higher values. Additionally, this could be affected by the silica filler, leading to inhomogeneous charge distribution. The elemental analysis shows a higher amount of SiO_2_ than leftover PDMS. Thus, the more intense peak with the highest binding energy, at 104.3–104.6 eV, is assigned to fully oxidized Si, while the line at 103.4–103.7 eV is assigned to partially oxidized Si from not completely oxidized PDMS. Due to the different kinetic energies of silicon and oxygen photoelectrons, the information depth is 2–3 nm smaller for oxygen. The oxygen signal is formed by two peaks at 532.4–532.5 eV and 533.2–533.3 eV [59]. The former peak can be attributed to SiO_x_/SiO_2_, and the latter can be attributed either to OH groups, the charged PDMS layer or both. The relative reduction of the 533.2–533.3 peak can be explained by the condensation of the OH groups to Si-O-Si bonds or by an increase in the SiO_x_/SiO_2_ layer thickness. The smaller decrease observed between the samples treated for 60 and 75 min, as well as between the samples treated for 75 and 90 min, could be due to an inhomogeneous distribution of OH groups. This would also explain the difference in contact angle and bonding for which a homogeneous distribution of OH groups is necessary. However, the lower bonding stability of the 90 min treated sample compared to the 75 min treated sample can be explained by the condensation of surface OH groups or surface hardening due to a thicker SiO_x_/SiO_2_ layer.

### 3.2. Functionality of Microfluidic Chips Prepared Using UVO Bonding

**Figure 17:**
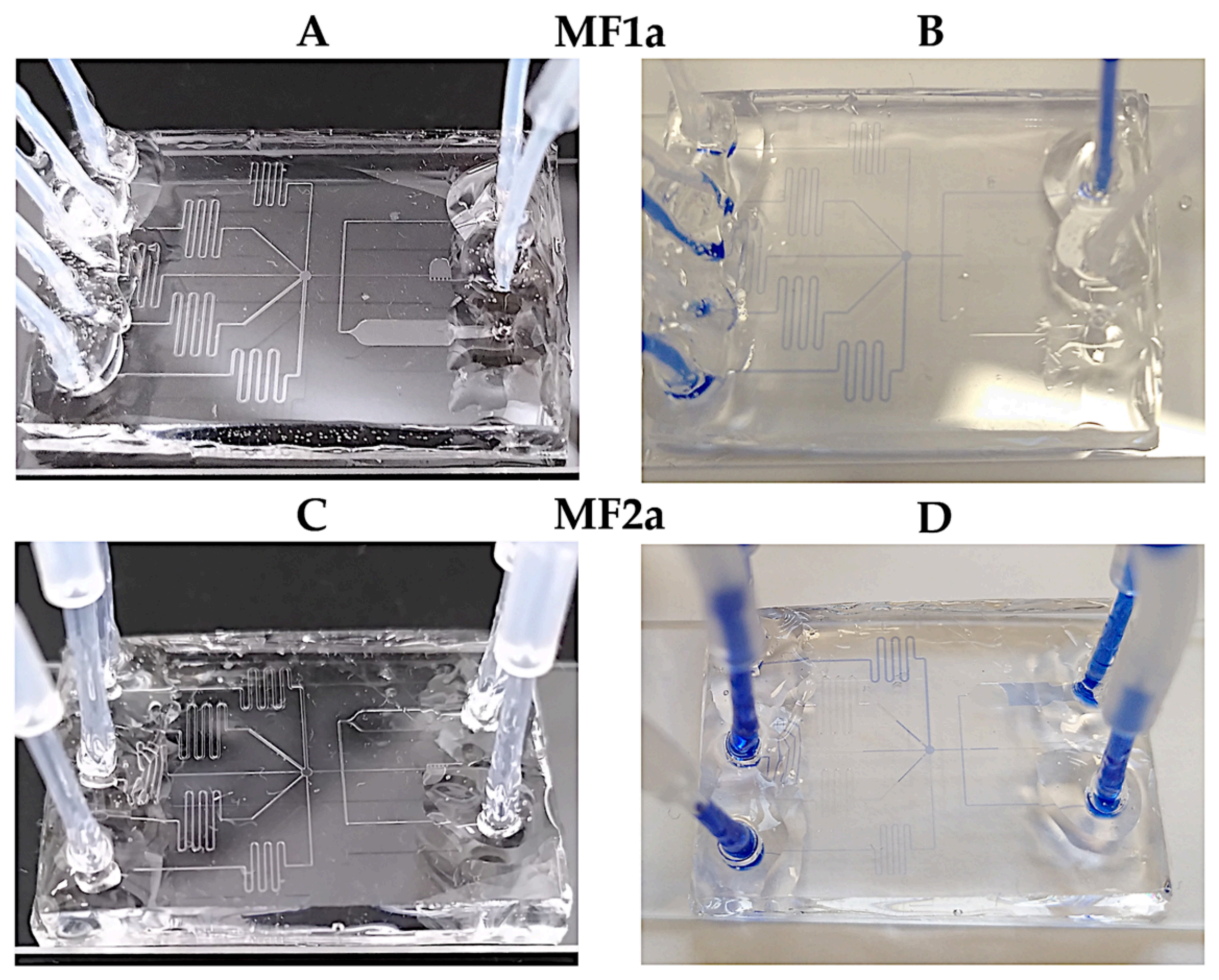
Exemplary photographs of the prepared 24 × 34.5 mm microfluidic chips MF1a (**top**) and MF2a (**bottom**): on the (**left**), after the tubes had been glued on; on the (**right**), in use with blue ink. In the case of MF1a (**A**,**B**), the bottom left tube had fallen off during gluing, and the inlet became clogged with glue, rendering it unusable.

Microfluidic chips MF1a–c and MF2a–c were prepared to serve as a proof of principle that functional microfluidic chips can be realized when using a UVO treatment. Figure 17 shows exemplary MF1a and MF2a and Figure S12 the other chips. In this case, no predetermined breaking structures were used, since the chips were not intended for long-term use. The gluing of the tubes was found to be sensitive to manual handling errors. For example, in the case of MF1a, the bottom left tube fell to the side while gluing the middle tube, and the glue clogged the corresponding inlet. However, no problem occurred during assembly of the other microfluidic chips.

**Figure 18:**
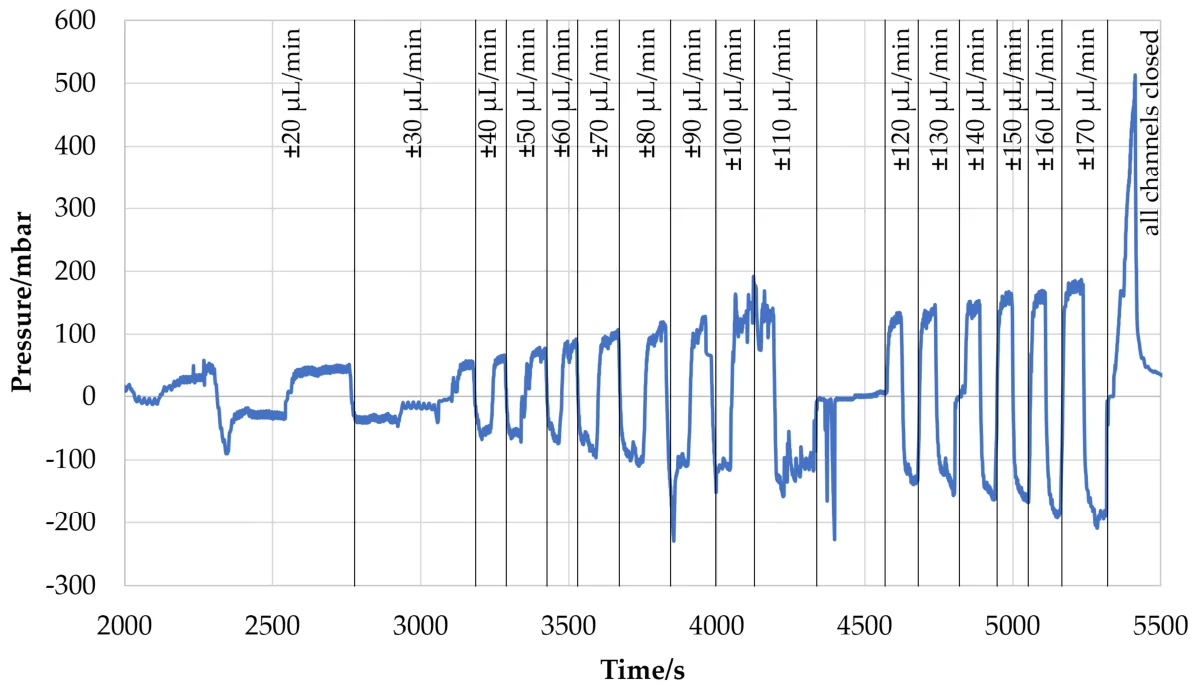
Results of the pressure measurement as a function of time and the different flow values used to prove functionality and the leak test as well as burst test (starting at ~5200 s) of MF1a.

The chips were used in a leakage test involving blue-ink-colored water. There were no internal bridges observable between the channels, which would indicate poor bonding due to insufficient activation of the surface. The pressure measurement for the chip MF1a is exemplarily, shown in Figure 18, and the measurements for MF2a, MF1b–c and MF2b–c are given in the Supplementary Materials in Figures S13 and S14, respectively. All chips could be used in vacuum- and pressure-driven systems, when they were connected to a stepper pump capable of generating a flow rate of 170 µL/min and applying, on average, a maximum operation pressures of 146 ± 50 mbar and −142 ± 41 mbar for the different chips. The differences in the pressure can be attributed to slight variations in the connections of the different chips. The average burst pressure in water is 428 ± 60 mbar.

The tubes were then closed with fold-back clamps for the burst test. The pressure increased up to a point when a leak appeared to determine the maximum possible pressure. In all cases, leakage occurred when the bonding broke across a wider area of the two layers, as exemplarily observable in the photographs provided in Supplementary Materials Figure S15. This is lower than for microfluidic chips prepared using oxygen plasma bonding, which we were able to operate up to at least 900 mbar. This is consistent with the weaker bonding strength described by Yousuff et al. [34]. The difference between the water and air measurements is known and can be attributed to bond hydrolysis due to water [25]. Nevertheless, this range is suitable for typical laboratory tests.

## 4. Conclusions

In conclusion, we presented a protocol for preparing microfluidic chips that uses the UV ozone cleaner by Ossila for bonding instead of oxygen plasma bonding. Table 7 shows a summary of the optimization results. The process is limited to PDMS@PDMS bonding, rather than PDMS@glass bonding or membranes. The problems caused by the hardening of the surface were overcome. It was used 24 times (usually for two microfluidic chips at the same time), and it led to fully bonded PDMS layers in 20 cases (~83%). Problems occurred when the samples were not attached immediately after the activation, when the after-treatment was too short, when the two layers were pressed together with too much force or when the layers were bent too much. The latter two factors both lead to cracks in the activated surface and, therefore, leakage. Additionally, we tested dividing the treatment into two parts with the same total treatment time to better accommodate working schedules, which is possible. Gluing tubes or valves with pre-polymerized PDMS is very reliable, and we have not experienced any problems with it so far as long as the chip is handled carefully during gluing to prevent pulling out inserted items.

Although this process is slower in comparison to oxygen plasma bonding, it is carried out using a less expensive, easier-to-handle instrument. This can be a viable alternative when only small quantities of microfluidic chips are required and an oxygen plasma etcher is unavailable. This is of interest to research facilities and fundamental researchers in the field of developing new systems and prototypes. Personnel costs would not exceed the cost of a plasma etching system, and the ability to individually control the test setups would allow one to always stay below 0.5 mbar pressure. However, this protocol is not suitable for preparing large batches of microfluidic chips because the time required would lead to high personnel costs. In order to gain a broader understanding of UVO bonding in general, it would be interesting to compare the bonding processes, geometries and lamp intensities of different instruments in order to determine the most effective instrument and bonding conditions. However, this cannot be achieved using the limited information available in the existing literature, and it cannot be achieved with our instrument, for which we cannot set a certain temperature or vary and measure the light intensity.

## 5. Patents

The authors declare that a patent has been filed for the predetermined breaking structures and sealing process of the tubes under the number (DE10 2026 109 856).

## Figures and Tables

**Table 1:** List of the mechanical parameters of PDMS used for the static analysis.

Parameter	Value	Explanation or Reference
Behavior	isotropic	Same as for glass
Thermal conductivity	0.27 W/m×K	Supplier data sheet
Specific heat	0.586 J/(g×K)	[52]
Thermal expansion	340 ppm/°C	Supplier data sheet
Elasticity modulus	2.5 MPa	[53]
Poisson’s ratio	0.5	[53]
Thrust module	0.83 MPa	[53]
Density	1.03 g/cm^3^	Supplier data sheet
Damping coefficient	0.02	[53]
Yield strength	5.9 MPa	[53]
Tensile strength	7.6 MPa	[53]

**Table 2:** The PDMS@glass bonding conditions were evaluated in four categories: no bonding (colorless); less than half of the area was bonded, and the two layers could be separated with minimal force (yellow); more than half of the area was bonded, and the two layers could only be separated by destroying the PDMS (blue); the whole area was bonded, and the two layers could only be separated by destroying the PDMS (green).

Position	Activation Time of Glass/Activation Time of PDMS									
	min	min	min	min	min	min	min	min	min	min
top left	20/10	25/15	30/20	60/10	60/15	60/20	60/60	70/70	80/80	90/90
top right	20/10	25/15	30/20	60/10	60/15	60/20	60/60	70/70	80/80	90/90
middle left	20/10	25/15	30/20	60/10	60/15	60/20	60/60	70/70	80/80	90/90
middle	20/10	25/15	30/20	60/10	60/15	60/20	60/60	70/70	80/80	90/90
middle right	20/10	25/15	30/20	60/10	60/15	60/20	60/60	70/70	80/80	90/90
bottom left	20/10	25/15	30/20	60/10	60/15	60/20	60/60	70/70	80/80	90/90
bottom right	20/10	25/15	30/20	60/10	60/15	60/20	60/60	70/70	80/80	90/90

**Table 3:** The PDMS@PDMS bonding conditions were evaluated in four categories: no bonding (colorless); less than half of the area was bonded, and the two layers could be separated with minimal force (yellow); more than half of the area was bonded, and the two layers could only be separated by destroying the PDMS (blue); the whole area was bonded, and the two layers could only be separated by destroying the PDMS (green).

Position	Activation Time of PDMS (Same for Both Sides)									
	min	min	min	min	min	min	min	min	min	min
top left	10	15	20	30	40	50	60	70	-	-
top right	10	15	20	30	40	50	60	70	-	-
middle left	10	15	20	30	40	50	60	70	-	-
middle	10	15	20	30	40	50	60	70	80	90
middle right	10	15	20	30	40	50	60	70	-	-
bottom left	10	15	20	30	40	50	60	70	-	-
bottom right	10	15	20	30	40	50	60	70	-	-

**Table 4:** Maximum pressure values determined in the air burst test of the simplified inlet samples bonded after 60–90 min UVO treatment (T_Start_ ≈ 25 °C). Reasons for the damage: (a) detachment or the PDMS layers, (b) detachment of the glue, (c) slight delamination of the bonded PDMS layers around the inlets without a leakage after the 10 min test and (d) delamination of glue during the 10 min test.

Sample Nr.	60 min	70 min	75 min	80 min	90 min
1	>1081	547 ^a^	>1027 ^a,b^	550 ^a^	596 ^a^
2	680 ^a^	548 ^a^	>1107 ^b^	>1053 ^d^	>1106 ^b^
3	695 ^a^	>1086	>1087	853 ^a^	>1142
4	788 ^a^	>1010 ^c^	>1086 ^a,b^	>1120 ^b^	>1123
5	562 ^a^	560 ^a^	>1137 ^c^	489 ^a^	885 ^a^
Average	761	750	>1000	813	970
Standard deviation	196	273	-	286	234

**Table 5:** Elemental composition at the surface of 60 min, 75 min and 90 min UVO-treated PDMS as determined from the XPS high-resolution scans.

at%	C–C 1s	O–O 1s	Si–Si 2p
PDMS (theoretical)	50	25	25
Untreated PDMS	43	28.1	28.9
60 min UVO-treated PDMS	13	55.8	31.2
75 min UVO-treated PDMS	11.5	57.2	31.3
90 min UVO-treated PDMS	10.4	58.6	31
Glass substrate	11	57.9	31.1
30 SiO_2_ + 11 C + 2 Si	11.7	56.9	31.4

**Table 6:** Comparison of the XPS measurement results with those in the literature. The corresponding atoms to the peaks are highlighted in bold.

Peak Positions (C at 284.4 eV)	Peak Positions (C at 284.8 eV)	Attributed to	Literature Values	Refs.
eV	eV		eV	
101.1	101.5	**Si**-C/O (CH_3_)_3_SiO_1/2_)	101.6	[58]
102.4	102.7	**Si**-C/O (CH_3_SiO_3/2_)	102.7	[58]
284.4	284.8	Si-**C**	284.4	[58,59]
285.2	285.6	**C**-O or **C**-**C** (impurity)	286.3–286.5/284.8–285	[60]
531.2	531.6	Si-**O** ((CH_3_)_2_SiO_2_)	532.1	[58]
532	532.3	Si-**O** ((CH_3_)_3_SiO_1/2_) (CH_3_SiO_3/2_))	532.2/532.3	[58]

**Table 7:** Summary of measurement results.

Measurement	Result
Contact angle measurement	UVO treatment time > 60 min
Peel test	UVO treatment time > 70 min
Burst pressure (air)	>1000 mbar (75 min UVO treatment time)
Tube connection	Use of PDMS seals (0.4 mm thickness and 3–4 mm outer diameter) and pre-polymerized PDMS glue
Burst pressure (water)	428 ± 60 mbar (70 min UVO treatment time)

## Data Availability

The data supporting this article and the DWG files of the structures have been included as part of the Supplementary Materials.

## Supplementary Materials

The following supporting information can be downloaded at: https://www.mdpi.com/article/10.3390/mi17091050/s1, Table S1: Overview of the different conditions described in the literature for UVO bonding. n.p. = not provided (there is no guarantee of completeness). Table S2: Micrographs from the mechanical stability test. Each structure was prepared and tested four times. For all pictures, contrast and sharpness were increased to increase the visibility of the fine cracks. Figure S1: Left: Schematic overview of the most important factors for creating a microfluidic chip that are discussed within this study. Right: Photograph of the UV ozone cleaner by Ossila. Figure S2: Exemplary photographs of the contact angle measurement of the glass and PDMS samples a few minutes after 10–70 min UVO treatment. Figure S3: Pictures of cracks at the bonded surfaces of a microfluidic chips when applying punctual pressure. Figure S4: Design of the inlets with predetermined breaking structures. Figure S5: Top: Inlets with surrounding predetermined breaking structures before (top) and after inserting tubes (bottom). In case A, the hole was prepared with a 1 mm biopsy punch, and the tube has 1 mm OD. In cases B, C and D, the hole was punched with a 1.5 mm puncher, and the tube has an OD of 1.6 mm. For all pictures, contrast and sharpness were increased to increase the visibility of the fine cracks. Figure S6: Example of an inlet with the 15 ring areas in red for the evaluation of the cracks and areas of broken bonding. Figure S7: Bodies for the static simulation of inlets with predetermined breaking structures (top) and layered PDMS with two or five different elastic moduli (bottom). Figure S8: Left: Examples of micrographs of tubes glued with pre-polymerized PDMS (in this case, colored with carbon black to make the PDMS glue and the seals more easily visible). In all cases, the PDMS glue got between the wall and the tube but did not reach the end of the tube. Even though the PDMS was colored with carbon black, it was difficult to see the glue, which is why manual handling of the seals was chosen as an exclusion criterion instead of the most effective way of preventing PDMS from getting in. Figure S9: Assembly of the samples for the burst test. Figure S10: Measurement data of the burst test in air of five simplified bonding samples within the range of 60–90 min UVO treatment time. The maximum pressure for the five samples is marked above the measurement curve for easier readout. Figure S11: Measurement data for the long-term measurement of the samples that were not damaged during the first burst test. Decreasing pressure can be attributed to no fully gas-tight materials, and in some of the cases, it is due to the rotor position of the peristaltic pump. Figure S12: Photographs of MF1b, MF1c, MF2b and MF2c after preparation and during the operation with diluted ink. Figure S13: Results of the pressure measurement as a function of time and the different flow values used to prove functionality of MF2. The larger scattering in comparison to the results for MF1 is due to air bubbles in the tubes and the MF chip. Figure S14: Results of the pressure measurement as a function of time and the different flow values used to prove functionality of MF1b, MF1c, MF2b and MF2c. Spikes in the MF1b measurement can be attributed to bubbles within the system. Figure S15: MF1 (left) and MF2 (right) after the burst test. In both cases, the leakage (left) and the area where the bonding was broken due to the high pressure (colored with blue ink) can be seen (right).

## Abbreviations

The following abbreviations are used in this manuscript:

DI	Deionized
PDMS	Polydimethylsiloxane
UVO	UV ozone
MF	Microfluidic chip
PGMEA	1-Methoxy-2-propylacetat
ID	Inner diameter
OD	Outer diameter
