# Supplementary material for: Bonding with a Benchtop UV Ozone Cleaner: A Practical Guide to Prepare a Microfluidic Chip
Source: Micromachines (Basel). 2026 Sep 2;17(9):1050. doi: 10.3390/mi17091050 (PMC13609066; doi:10.3390/mi17091050)
Supplement: Supplementary file 1 [file micromachines-17-01050-s001.zip › micromachines-4538803-supplementary.pdf]

Bonding with a Bench-Top UV Ozone Cleaner – A Practical Guide to Prepare a Microfluidic Chip

Supporting information

Mirjam Poschmann: Chair for Integrated Systems and Photonics, Faculty of Engineering, Kiel University, Kaiserstraße 2, 24143 Kiel, Germany, mip@tf.uni-kiel.de

Lucas Holtorf: Chair for Integrated Systems and Photonics, Faculty of Engineering, Kiel University Kaiserstraße 2, 24143 Kiel, Germany, lho@tf.uni-kiel.de

Igor Titov: Chair for Integrated Systems and Photonics, Faculty of Engineering, Kiel University Kaiserstraße 2, 24143 Kiel, Germany, igti@tf.uni-kiel.de

Nahomy M. Lizarde: Chair for Composite Materials, Faculty of Engineering, Kiel University, Kaiserstraße 2, 24143 Kiel, Germany

Thomas Strunskus: Chair for Composite Materials, Faculty of Engineering, Kiel University, Kaiserstraße 2, 24143 Kiel, Germany, ts@tf.uni-kiel.de

Martina Gerken: Chair for Integrated Systems and Photonics, Faculty of Engineering, Kiel University Kaiserstraße 2, 24143 Kiel, Germany, mge@tf.uni-kiel.de

Tables

Table S1 Overview of the different conditions described in the literature for UVO bonding. n.p. = not provided (there is no guarantee of completeness). 3
Table S2 Micrographs from the mechanical stability test. Each structure was prepared and tested four times. For all pictures, contrast and sharpness were increased to increase the visibility of the fine cracks. 5

Figures

Figure S1 Left: Schematic overview of the most important factors for creating a microfluidic chip that are discussed within this study. Right: Photograph of the UV ozone cleaner by Ossila. 3
Figure S2 Exemplary photographs of the contact angle measurement of the glass and PDMS samples a few minutes after 10-70 min UVO treatment. 4
Figure S3 Pictures of cracks at the bonded surfaces of a microfluidic chips when applying punctual pressure. 4
Figure S4 Design of the inlets with predetermined breaking structures. 4
Figure S5: Inlets with surrounding predetermined breaking structures before (top) and after inserting tubes (bottom). In case A, the hole was prepared with a 1 mm biopsy punch, and the tube has 1 mm OD. In cases B, C and D, the hole was punched with a 1.5 mm puncher, and the tube has a OD of 1.6 mm. For all pictures, contrast and sharpness were increased to increase the visibility of the fine cracks. 4
Figure S6 Example of an inlet with the 15 ring areas in red for the evaluation of the cracks and areas of broken bonding. 6
Figure S7 Bodies for the static simulation of inlets with predetermined breaking structures (top) and layered PDMS with two or five different elastic moduli (bottom). 7
Figure S8 Left: Examples of micrographs of tubes glued with pre-polymerized PDMS (in this case, colored with carbon black to make the PDMS glue and the seals more easily visible). In all cases, the PDMS glue got between the wall and the tube, but did not reach the end of the tube. Even though the PDMS was colored with carbon black, it was difficult to see the glue, which is why manual handling of the seals was chosen as an exclusion criterion instead of the most effective way of preventing PDMS from getting in. 7
Figure S9 Assembly of the samples for the burst test. 7
Figure S10 Measurement data of the burst test in air of five simplified bonding samples within the range of 60-90 min UVO treatment time. The maximum pressure for the five samples is marked above the measurement curve for easier readout. 8
Figure S11 Measurement data for the long-term measurement of the samples that were not damaged during the first burst test. Decreasing pressure can be attributed to no fully gas-tight materials, and in some of the cases, it is due to the rotor position of the peristaltic pump. 8
Figure S12 Photographs of MF1b, MF1c, MF2b and MF2c after preparation and during the operation with diluted ink. 8
Figure S13 Results of the pressure measurement as a function of time and the different flow values used to prove functionality of MF2. The larger scattering in comparison to the results for MF1 is due to air bubbles in the tubes and the MF chip. 9
Figure S14 Results of the pressure measurement as a function of time and the different flow values used to prove functionality of MF1b, MF1c, MF2b and MF2c. Spikes in the MF1b measurement can be attributed to bubbles within the system. 9
Figure S15 MF1 (left) and MF2 (right) after the burst test. In both cases, the leakage (left) and the area where the bonding was broken due to the high pressure (colored with blue ink) can be seen (right). 9

Table S1 Overview of the different conditions described in the literature for UVO bonding. n.p. = not provided (there is no guarantee of completeness).

			Materials		activation	aftertreatment		Cit.
Nr	Instrument	lamp intensity	layer	substrate	t	T	t	
					min	°C	min	
1	n. p.	n. p.	PDMS	glass	n. p.	120	300	[1]
2	Electro-Technic Products Inc. model BD-20)	n. p.	PDMS	PDMS	n. p.	80	10	[2]
3	n. p.	n. p.	PDMS	glass	n. p.	75	30	[3]
4	n. p.	n. p.	PDMS	PSA	n. p.	n. p.	n. p.	[4]
5	n. p.	n. p.	PDMS	Silicone gaskets	15	70	120	[5]
6	n. p.	n. p.	PDMS	PDMS	n. p.	n. p.	n. p.	[6]
7	n. p.	n. p.	PDMS	PDMS	n. p.	120	over night	[7]
8	n. p.	n. p.	PDMS	PDMS/Ecoflex	3	70	12h	[8]
9	n. p.	n. p.	PDMS	glass	n. p.	n. p.	n. p.	[9]
10	n. p.	n. p.	PDMS	glass	40	n. p.	n. p.	[10]
11	n. p.	n. p.	PDMS	glass	40	n. p.	n. p.	[11]
12	n. p.	n. p.	PDMS	glass	5	n. p.	n. p.	[12]
13	n. p.	n. p.	PDMS	glass	n. p.	n. p.	n. p.	[13]
14	model 342, Jelight Co.	28-32 mW/cm² [14]	PDMS	PDMS/Ecoflex	30	120	20	[15]
15	n. p.	n. p.	PDMS	glass	40	n. p.	n. p.	[16]
16	Nova scan Inc. Ames, IA	n. p.	PDMS	Glass/PDMS	3-5	80	15	[17]

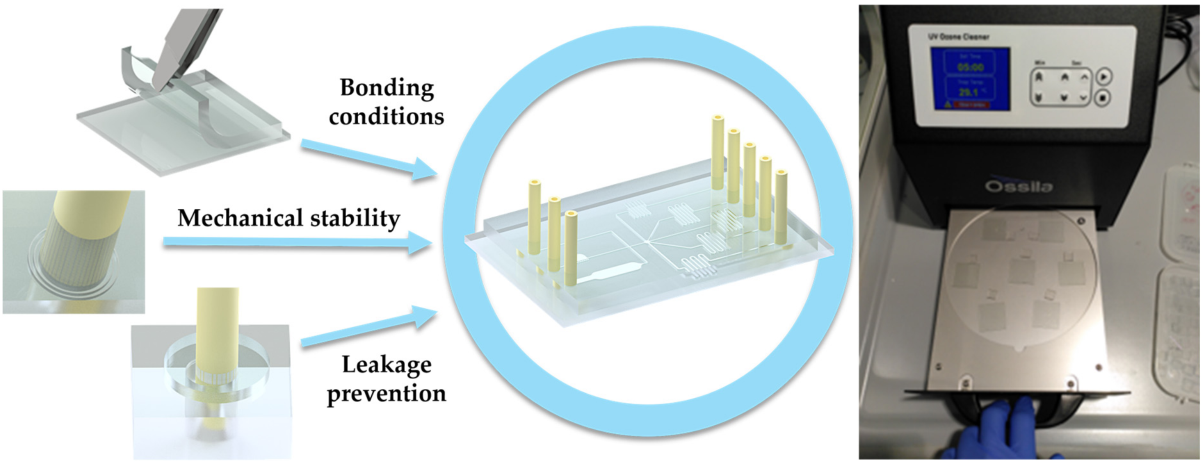

Figure S1 Left: Schematic overview of the most important factors for creating a microfluidic chip that are discussed within this study. Right: Photograph of the UV ozone cleaner by Ossila.

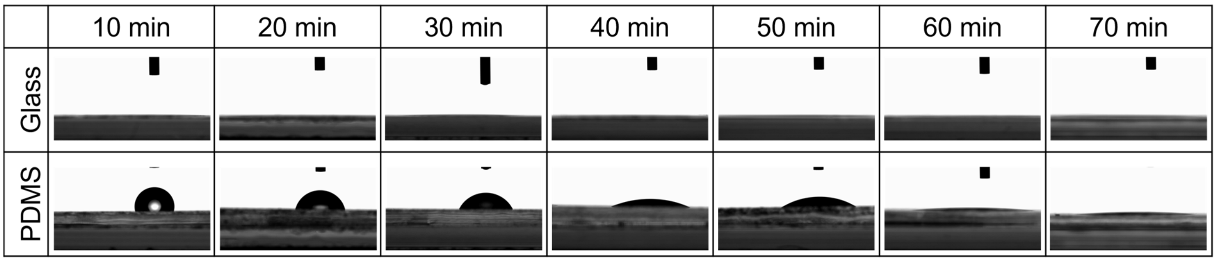

Figure S2 Exemplary photographs of the contact angle measurement of the glass and PDMS samples a few minutes after 10-70 min UVO treatment.

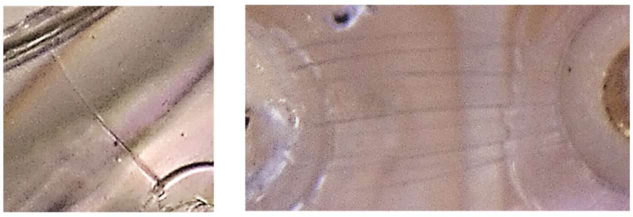

Figure S3 Pictures of cracks at the bonded surfaces of a microfluidic chips when applying punctual pressure.

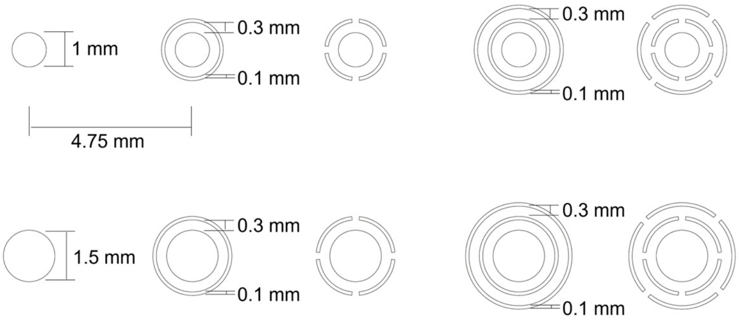

Figure S4 Design of the inlets with predetermined breaking structures.

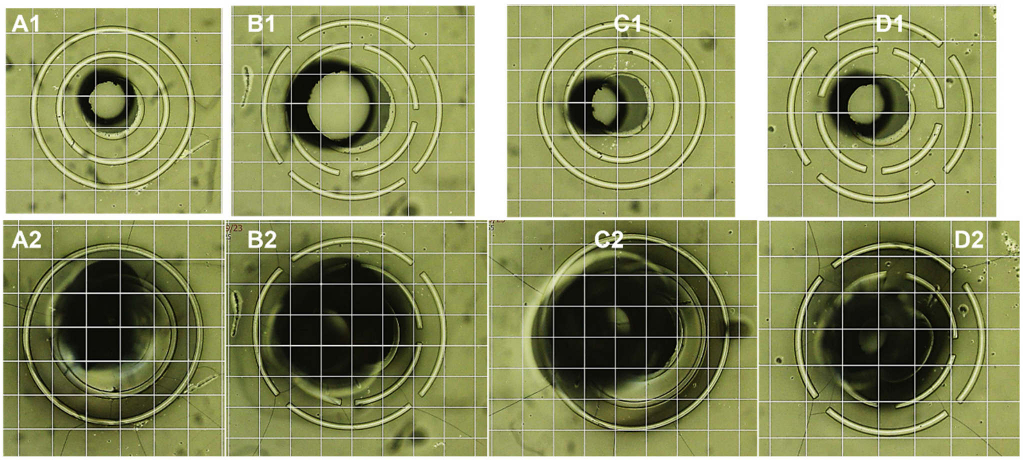

Figure S5: Inlets with surrounding predetermined breaking structures before (top) and after inserting tubes (bottom). In case A, the hole was prepared with a 1 mm biopsy punch, and the tube has 1 mm OD. In cases B, C and D, the hole was punched with a 1.5 mm puncher, and the tube has a OD of 1.6 mm. For all pictures, contrast and sharpness were increased to increase the visibility of the fine cracks.

Table S2 Micrographs from the mechanical stability test. Each structure was prepared and tested four times. For all pictures, contrast and sharpness were increased to increase the visibility of the fine cracks.

	Slight pressing and moving = usual handling	***Strong pressing and moving = investigate the weak spots***
1	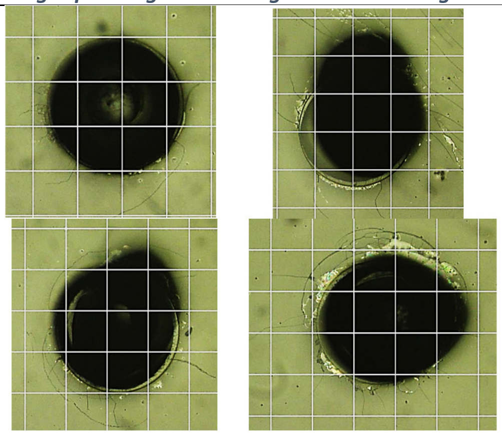	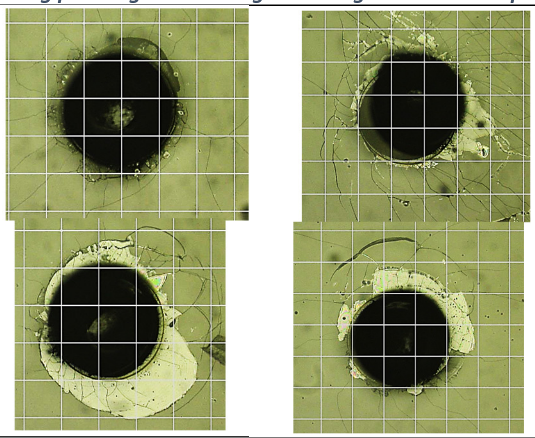
2	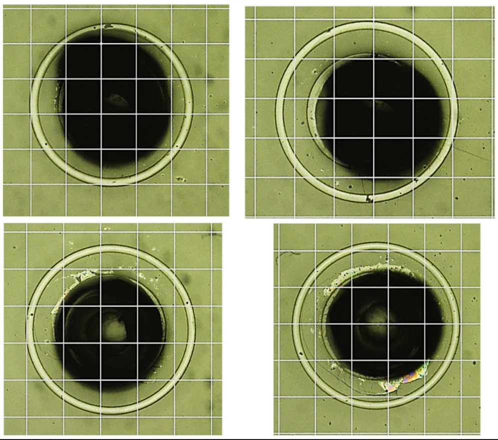	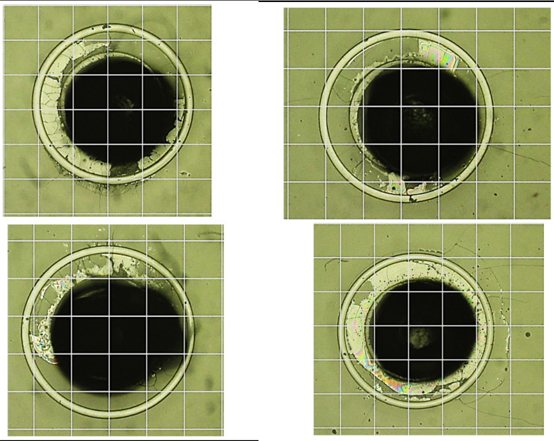
3	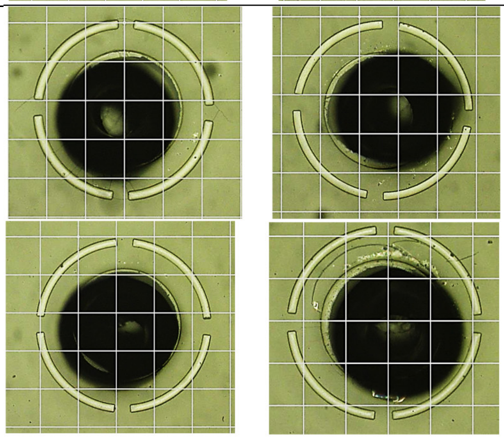	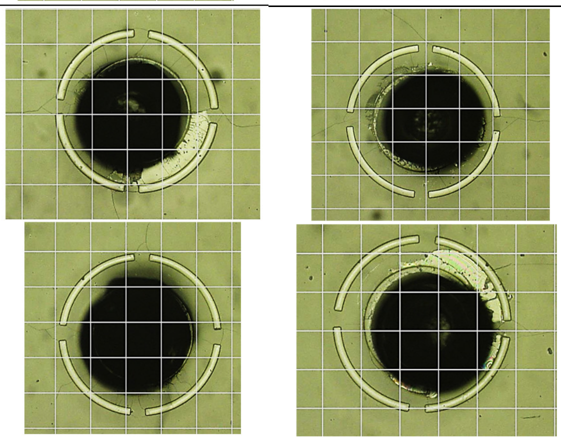
4	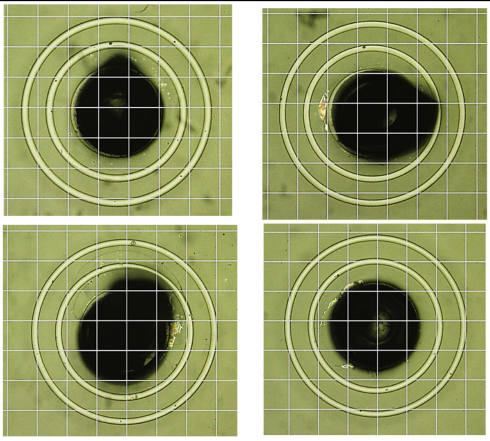	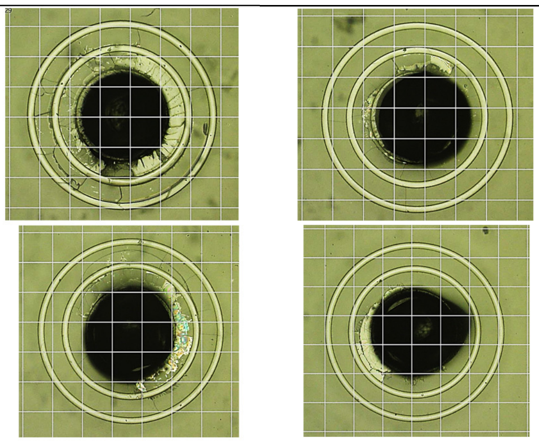

5	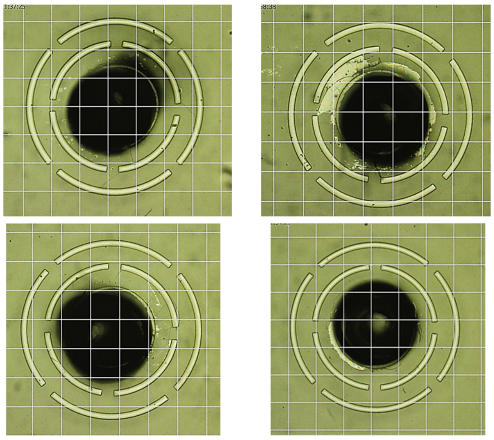	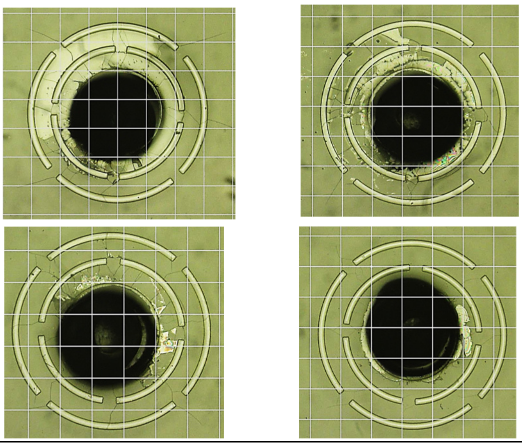

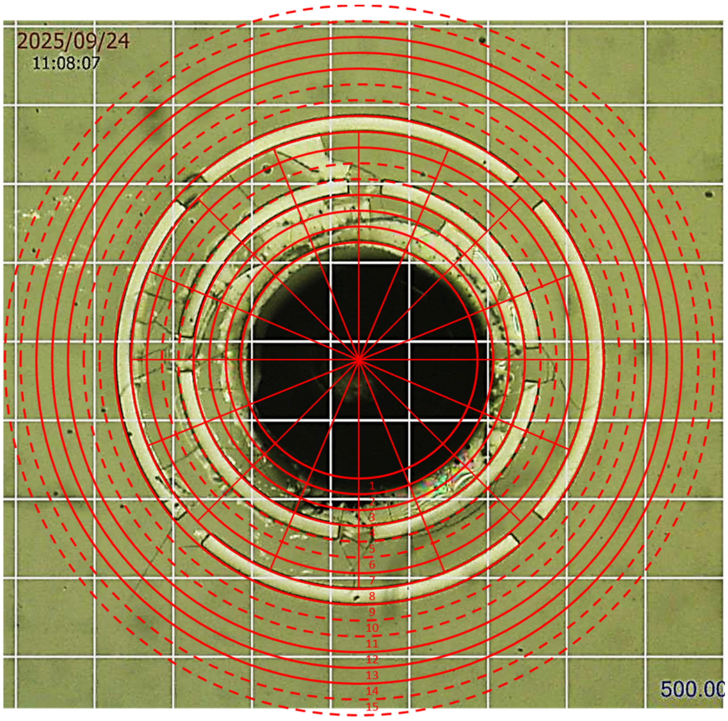

Figure S6 Example of an inlet with the 15 ring areas in red for the evaluation of the cracks and areas of broken bonding.

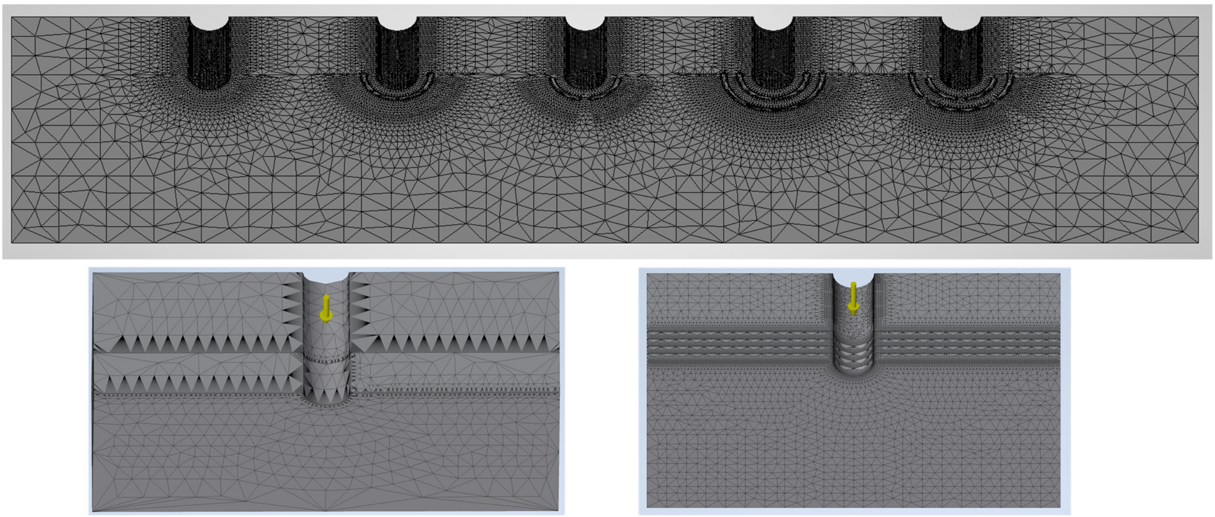

Figure S7 Bodies for the static simulation of inlets with predetermined breaking structures (top) and layered PDMS with two or five different elastic moduli (bottom).

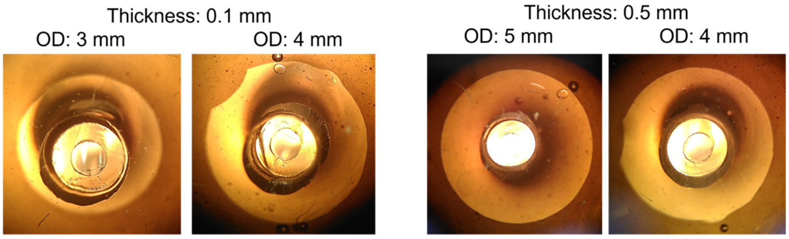

Figure S8 Left: Examples of micrographs of tubes glued with pre-polymerized PDMS (in this case, colored with carbon black to make the PDMS glue and the seals more easily visible). In all cases, the PDMS glue got between the wall and the tube, but did not reach the end of the tube. Even though the PDMS was colored with carbon black, it was difficult to see the glue, which is why manual handling of the seals was chosen as an exclusion criterion instead of the most effective way of preventing PDMS from getting in.

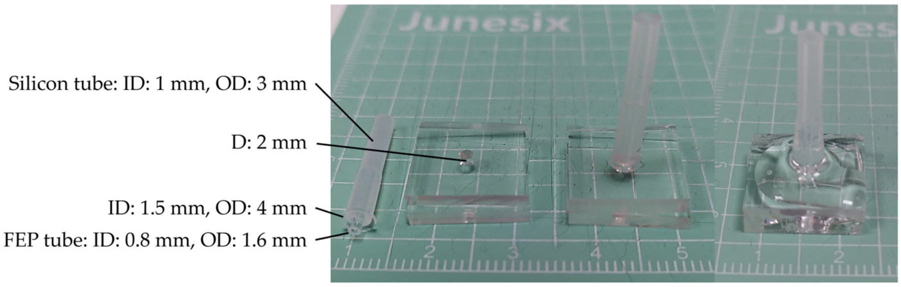

Figure S9 Assembly of the samples for the burst test.

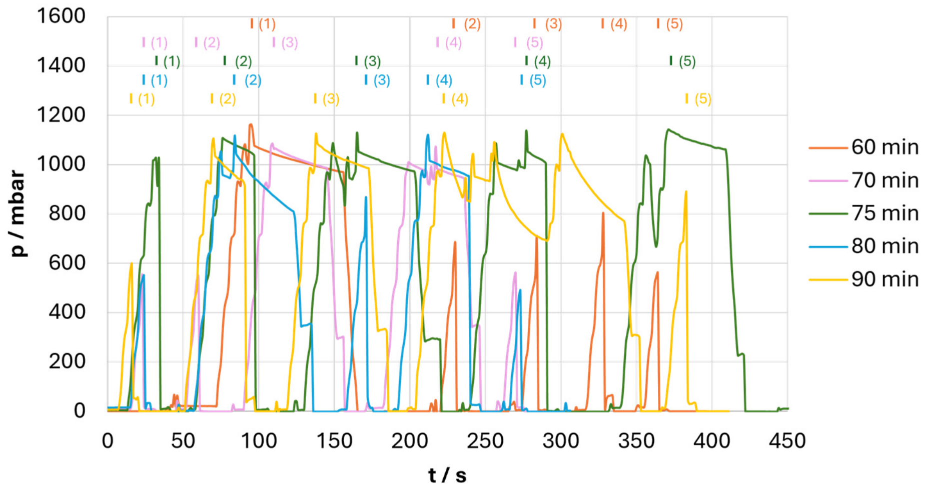

Figure S10 Measurement data of the burst test in air of five simplified bonding samples within the range of 60-90 min UVO treatment time. The maximum pressure for the five samples is marked above the measurement curve for easier readout.

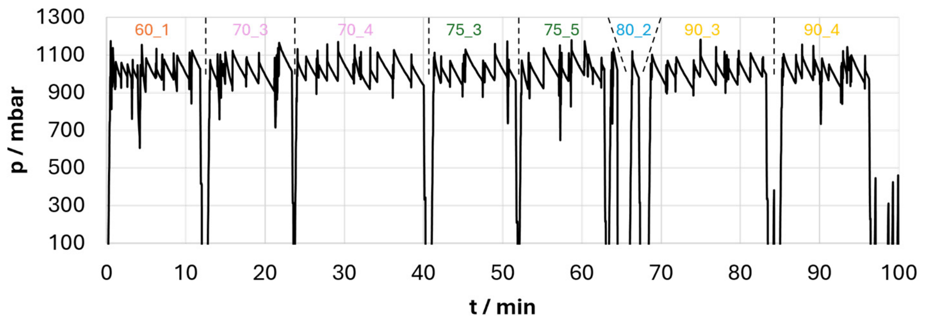

Figure S11 Measurement data for the long-term measurement of the samples that were not damaged during the first burst test. Decreasing pressure can be attributed to no fully gas-tight materials, and in some of the cases, it is due to the rotor position of the peristaltic pump.

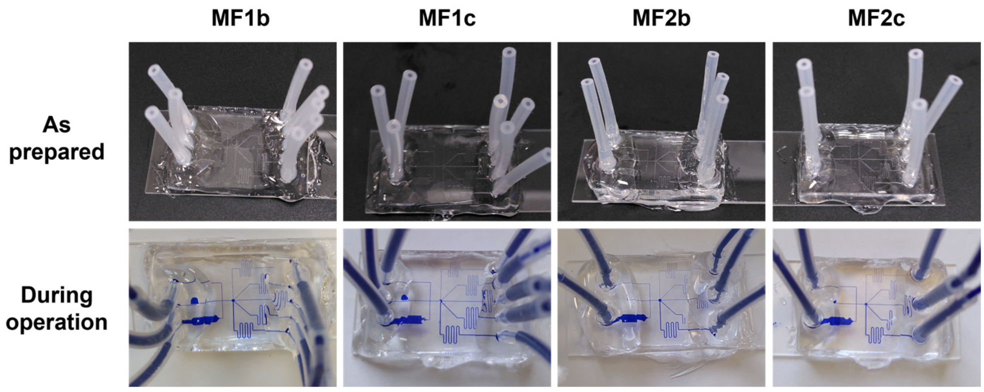

Figure S12 Photographs of MF1b, MF1c, MF2b and MF2c after preparation and during the operation with diluted ink.

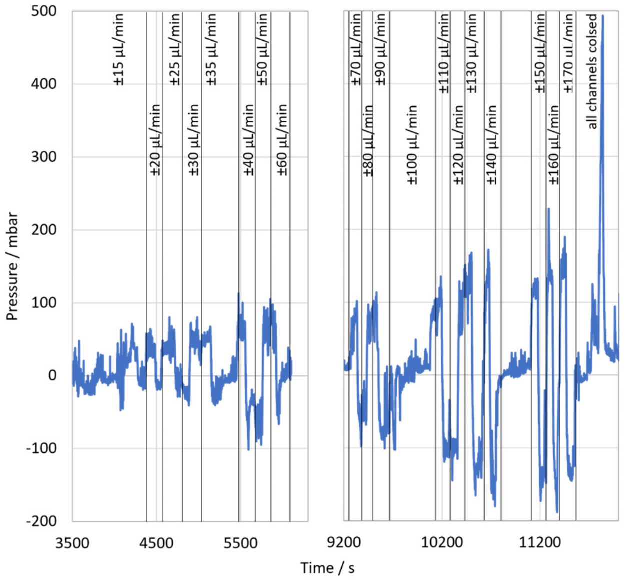

Figure S13 Results of the pressure measurement as a function of time and the different flow values used to prove functionality of MF2. The larger scattering in comparison to the results for MF1 is due to air bubbles in the tubes and the MF chip.

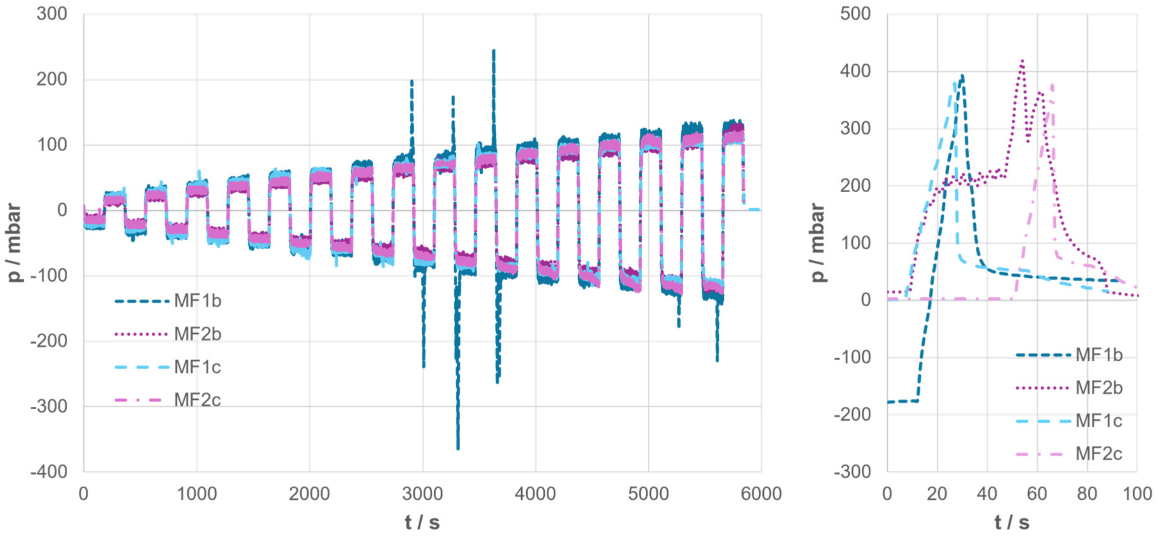

Figure S14 Results of the pressure measurement as a function of time and the different flow values used to prove functionality of MF1b, MF1c, MF2b and MF2c. Spikes in the MF1b measurement can be attributed to bubbles within the system.

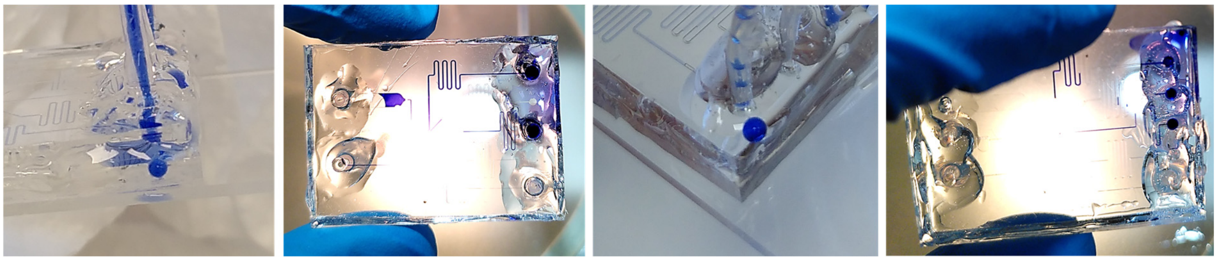

Figure S15 MF1 (left) and MF2 (right) after the burst test. In both cases, the leakage (left) and the area where the bonding was broken due to the high pressure (colored with blue ink) can be seen (right).

References

1. Bhardwaj, T.; Jha, S.K. On-Line PAT Monitoring of Lucentis in the Fermenter Using Aptamer-Based Capacitive Microfluidic Chip Biosensor. *J. Electrochem. Soc.* **2022**, *169*, 057512, doi:10.1149/1945-7111/ac5799.
2. Williams, M.C.; Wiens, C.; Hamilton, A.; Mancha, S.; Stalder, M.; Vargas, K.; Crawford, J.; Li, Y.; Schreiner, S.; Blake, D.J.; et al. Novel Fabrication Approach of a Porous Silicon Biocompatible Membrane Evaluated within a Alveolar Coculture Model 2021.
3. Bhardwaj, T.; Dalal, P.; Rathore, A.S.; Jha, S.K. An Aptamer Based Microfluidic Chip for Impedimetric Detection of Ranibizumab in a Bioreactor. *Sens. Actuators B Chem.* **2020**, *312*, 127941, doi:10.1016/j.snb.2020.127941.
4. Khire, T.S.; Salminen, A.T.; Swamy, H.; Lucas, K.S.; McCloskey, M.C.; Ajalik, R.E.; Chung, H.H.; Gaborski, T.R.; Waugh, R.E.; Glading, A.J.; et al. Microvascular Mimetics for the Study of Leukocyte–Endothelial Interactions. *Cel. Mol. Bioeng.* **2020**, *13*, 125–139, doi:10.1007/s12195-020-00611-6.
5. Mossu, A.; Rosito, M.; Khire, T.; Li Chung, H.; Nishihara, H.; Gruber, I.; Luke, E.; Dehouck, L.; Sallusto, F.; Gosselet, F.; et al. A Silicon Nanomembrane Platform for the Visualization of Immune Cell Trafficking across the Human Blood–Brain Barrier under Flow. *J. Cereb. Blood Flow Metab.* **2019**, *39*, 395–410, doi:10.1177/0271678X18820584.
6. Salminen, A.; Hill, K.; Henry Chung, L.; James McGrath, L.; Johnson, D.G. Protein Separation and Hemocompatibility of Nitride Membranes in Microfluidic Filtration Systems. In Proceedings of the 2018 40th Annual International Conference of the IEEE Engineering in Medicine and Biology Society (EMBC); July 2018; pp. 5814–5817.
7. Mansard, V.; Mecca, J.M.; Dermody, D.L.; Malotky, D.; Tucker, C.J.; Squires, T.M. Collective Rayleigh-Plateau Instability: A Mimic of Droplet Breakup in High Internal Phase Emulsion. *Langmuir* **2016**, *32*, 2549–2555, doi:10.1021/acs.langmuir.5b04727.
8. Kenry; Yeo, J.C.; Yu, J.; Shang, M.; Loh, K.P.; Lim, C.T. Highly Flexible Graphene Oxide Nanosuspension Liquid-Based Microfluidic Tactile Sensor. *Small* **2016**, *12*, 1593–1604, doi:10.1002/smll.201502911.
9. Dudu, V.; Able, R.A.; Rotari, V.; Kong, Q.; Vazquez, M. Role of Epidermal Growth Factor-Triggered PI3K/Akt Signaling in the Migration of Medulloblastoma-Derived Cells. *Cel. Mol. Bioeng.* **2012**, *5*, 402–413, doi:10.1007/s12195-012-0253-8.
10. Jha, S.K.; Joo, G.-S.; Ra, G.-S.; Lee, H.H.; Kim, Y.-S. Development of PCR Microchip for Early Cancer Risk Prediction. *IEEE Sens. J.* **2011**, *11*, 2065–2070, doi:10.1109/JSEN.2011.2105262.
11. Jha, S.K.; Jang, Y.-C.; Chand, R.; Islam, K. A PDMS Based Integrated PCR Microchip for Genetic Analysis.; 2011; pp. 273–275.
12. Ferguson, B.S.; Buchsbaum, S.F.; Swensen, J.S.; Hsieh, K.; Lou, X.; Soh, H.T. Integrated Microfluidic Electrochemical DNA Sensor. *Anal. Chem.* **2009**, *81*, 6503–6508, doi:10.1021/ac900923e.
13. Ra, G.-S.; Jha, S.K.; Yoon, T.-S.; Lee, H.H.; Kim, Y.-S. Improvement of Dynamic Characteristics of Polydimethylsiloxane Based Microvalve. *Microsyst. Technol.* **2009**, *15*, 607–609, doi:10.1007/s00542-008-0743-8.
14. Manuals – Jelight.
15. Chen, H.-Y.; McClelland, A.A.; Chen, Z.; Lahann, J. Solventless Adhesive Bonding Using Reactive Polymer Coatings. *Anal. Chem.* **2008**, *80*, 4119–4124, doi:10.1021/ac800341m.
16. Joung, S.-R.; Kang, C.J.; Kim, Y.-S. Series DNA Amplification Using the Continuous-Flow Polymerase Chain Reaction Chip. *Jpn. J. Appl. Phys.* **2008**, *47*, 1342, doi:10.1143/JJAP.47.1342.
17. Yousuff, C.M.; Danish, M.; Ho, E.T.W.; Kamal Basha, I.H.; Hamid, N.H.B. Study on the Optimum Cutting Parameters of an Aluminum Mold for Effective Bonding Strength of a PDMS Microfluidic Device. *Micromachines* **2017**, *8*, 258, doi:10.3390/mi8080258.
